# Sox9 is involved in the thyroid differentiation program and is regulated by crosstalk between TSH, TGFβ and thyroid transcription factors

**DOI:** 10.1038/s41598-022-06004-1

**Published:** 2022-02-09

**Authors:** Arístides López-Márquez, Carlos Carrasco-López, Andrea Martínez-Cano, Pascale Lemoine, Christophe E. Pierreux, Pilar Santisteban

**Affiliations:** 1 Instituto de Investigaciones Biomédicas “Alberto Sols”. Consejo Superior de Investigaciones Científicas (CSIC) y Universidad Autónoma de Madrid (UAM), CSIC-UAM., C/Arturo Duperier 4, 28029 Madrid, Spain; 2 Laboratorio de Investigación Aplicada en Enfermedades Neuromusculares, Unidad de Patología Neuromuscular, Servicio de Neuropediatría, Institut de Recerca Sant Joan de Déu, Esplugues de Llobregat, Barcelona, Spain; 3 Centro de Investigaciones Biomédicas en Red del Cáncer (CIBERONC), Instituto de Salud Carlos III, Madrid, Spain; 4 Cell Biology Unit, de Duve Institute and Université Catholique de Louvain, 1200 Brussels, Belgium

**Keywords:** Cell biology, Molecular biology, Endocrinology

## Abstract

While the signaling pathways and transcription factors involved in the differentiation of thyroid follicular cells, both in embryonic and adult life, are increasingly well understood, the underlying mechanisms and potential crosstalk between the thyroid transcription factors Nkx2.1, Foxe1 and Pax8 and inductive signals remain unclear. Here, we focused on the transcription factor Sox9, which is expressed in Nkx2.1-positive embryonic thyroid precursor cells and is maintained from embryonic development to adulthood, but its function and control are unknown. We show that two of the main signals regulating thyroid differentiation, TSH and TGFβ, modulate *Sox9* expression. Specifically, TSH stimulates the cAMP/PKA pathway to transcriptionally upregulate *Sox9* mRNA and protein expression, a mechanism that is mediated by the binding of CREB to a CRE site within the *Sox9* promoter. Contrastingly, TGFβ signals through Smad proteins to inhibit TSH-induced *Sox9* transcription. Our data also reveal that *Sox9* transcription is regulated by the thyroid transcription factors, particularly Pax8. Interestingly, Sox9 significantly increased the transcriptional activation of *Pax8* and *Foxe1* promoters and, consequently, their expression, but had no effect on *Nkx2.1*. Our study establishes the involvement of Sox9 in thyroid follicular cell differentiation and broadens our understanding of transcription factor regulation of thyroid function.

## Introduction

The thyroid is an endoderm-derived gland whose main function is the synthesis and secretion of the thyroid hormones T3 (triiodothyronine) and T4 (thyroxine), which are essential for the normal growth, differentiation and metabolism of several organs. Unlike other endoderm-derived organs, such as pancreas and liver, some aspects of the embryonic development of the thyroid gland remain unclear. During embryogenesis in the mouse, a small group of cells from the primitive pharyngeal endoderm simultaneously begin to express the so-called thyroid transcription factors Nkx2.1, Pax8 and Foxe1 at embryonic day (E) 8.5, a process known as specification. After annexing cells from the adjacent endoderm, these precursor cells migrate to their final destination just above the trachea, where they form follicles and express the necessary thyroid-specific genes for thyroid hormone production^1–4^. Studies in animal models and in pluripotent stem cells have contributed to a better understanding of the thyroid specification control. Besides leaded to the identification of several secreted growth factors important for thyroid development, such as Sonic Hedgehog^5^, fibroblast growth factor (Fgf)2, bone morphogenic protein 4^6^ and Fgf10^7^, which are derived mostly from the surrounding cardiogenic mesoderm^8,9^. Likewise, new transcription factors have been identified that act upstream or cooperate with the thyroid transcription factors during embryonic thyroid development, supporting the existence of a complex network of pathways involved in thyroid differentiation^2^. Among them, the transcription factor Sox9 has recently emerged as a critical regulator of early thyroid development^7^.

Sox9 belongs to the Sox family of transcriptional regulators (Sry-related HMG Box)^10–12^ and is involved in the development of several endoderm-derived organs including pancreas and liver^4,13–15^. Together with Fgf10, Sox9 controls thyroid morphogenesis by regulating the branching process during the expansion of the thyroid anlage^7^. This process is conserved in other organs such as the lung and exocrine glands^13,16–19^. These findings have provided a more complete picture of the mechanisms controlling the growth and expansion of thyroid precursors during thyroid development. *Sox9* is expressed at E9.5 in mice, and remains expressed even at later stages of thyroid embryogenesis where it marks a group of proliferating thyroid precursors involved in thyroid branching growth. It has been reported that various tissues including liver, pancreas and intestine contain a subset of *Sox9*-expressing cells with regenerative capacity during adulthood^20^, reinforcing the important role of Sox9 in sustaining the undifferentiated state of cell precursors during embryonic development and adulthood, including possibly the thyroid^7,21^. Furthermore, given that *Sox9* is expressed before terminal differentiation of the thyroid follicular cells, and that its expression is maintained in the adult thyroid, it can be hypothesized that this factor likely functions in the transcriptional network that regulates thyroid differentiation and function.

In addition to the thyroid transcription factors, several hormones and growth factors regulate follicular cell differentiation. Among them, thyrotropin (TSH)^22^ and transforming gowth factor (TGFβ)^23,24^ appear to play critical roles. Acting through their respective main signaling pathways, cAMP/CREB and Smad, both ligands regulate the expression of thyroid differentiation markers such as Pax8^25,26^, Foxe1^27^, thyroglobulin (Tg)^28,29^, thyroperoxidase (TPO)^24,30^ and the sodium iodide symporter (NIS)^31^.

Given the expression of *Sox9* in the embryonic thyroid and its potential to regulate the differentiation of thyroid follicular cells, we studied the role of Sox9 in thyroid follicular cell development, its control by the main regulators of thyroid function, TSH and TGFβ, and its involvement in the transcriptional network that controls thyroid differentiation and homeostasis.

## Results

### *Sox9* is expressed both in embryonic thyroid precursors and in adult thyroid follicular cells

**Figure 1:**
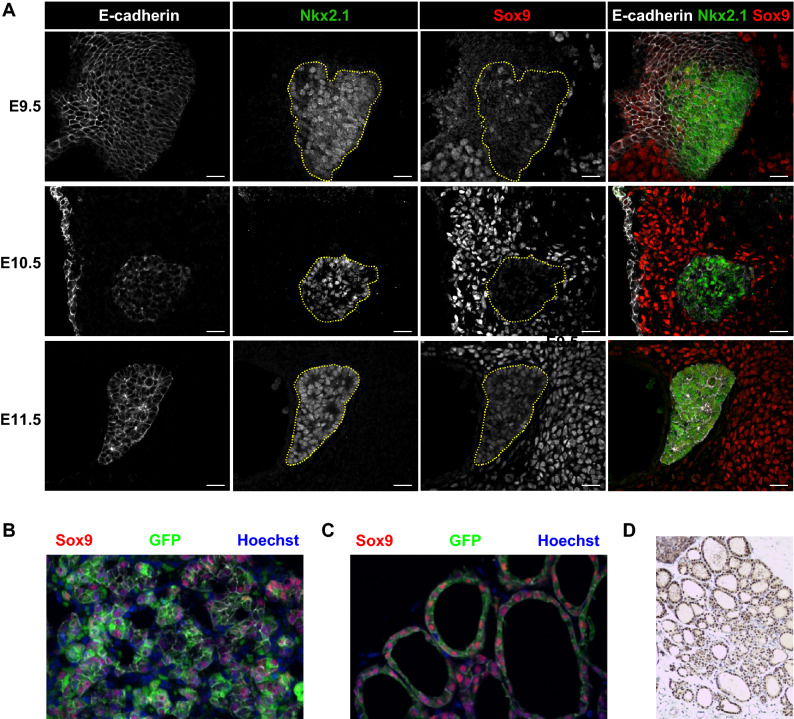
Immunolocalization of Sox9 in embryonic and adult mouse thyroid. (**A**) Sagittal sections of thyroid primordia at different stages of embryonic development immunolabeled for the transcription factors Nkx2.1 and Sox9, together with the epithelial marker cadherin (E-cadherin). The white arrows indicate the location of the thyroid primordium. (**B**) Thyroid sections from *Sox9-GFP* transgenic mouse embryos at E16.5 immunolabeled for Sox9 and GFP. Nuclei were counterstained with Hoechst. (**C**) Thyroid gland sections from *Sox9-GFP* transgenic adult mice immunolabeled for Sox9 and GFP. Nuclei were counterstained with Hoechst. (**D**) Thyroid gland sections from 2-month-old mice immunostained for Sox9 and counterstained with hematoxylin.

We first surveyed the expression of Sox9 in mouse thyroid sagittal sections at E9.5, E10.5 and E11.5 by immunofluorescence microscopy (Fig. 1A). We used Nkx2.1, a thyroid-specific marker^32^, to visualize the thyroid precursors emerging from the pharyngeal endoderm labeled with the epithelial-specific marker E-cadherin (Fig. 1A). We found that Sox9 was expressed weakly in a limited number of these thyroid precursors during early development, but was expressed at higher levels in the mesenchyme adjacent to the thyroid primordium. Sox9 expression increased in the thyroid primordium at later stages of embryonic development, and marked all the thyroid epithelial cells at E11.5. Sox9 expression in the mesenchyme surrounding the thyroid primordium was maintained along the different stages of embryonic development (Fig. 1A).

Because different members of the Sox family show a high similarity, especially Sox9 and Sox10^33^, we repeated the experiment in transgenic mice expressing green fluorescent protein (GFP), under the control of *Sox9* regulatory elements^34,35^ to unequivocally demonstrate that the signal detected during embryonic development is indeed Sox9. As shown in Fig. 1B, an intense GFP signal was evident in the thyroid of E16.5 embryos. Furthermore, immunofluorescence analysis of adult thyroid sagittal sections, from the same reporter mice, revealed robust Sox9 nuclear expression in the GFP^+^ epithelial cells forming follicles (Fig. 1C). These data demonstrate that Sox9 expression starts to be detected in the embryonic thyroid from the early budding stage, progressively increases as embryonic development continues, and is specifically found in follicular epithelial cells, confirming previous results^7^. Finally, we observed expression of Sox9 in the adult thyroid, as demonstrated by immunohistochemistry of thyroid sagittal sections in 2-month-old adult mice (Fig. 1D).

Overall, our results show that Sox9 is expressed in thyroid follicular cells during the middle and late embryonic stages, and is retained in the adult.

### TSH induces *Sox9* expression in thyroid follicular cells via the cAMP/PKA signaling pathway

**Figure 2:**
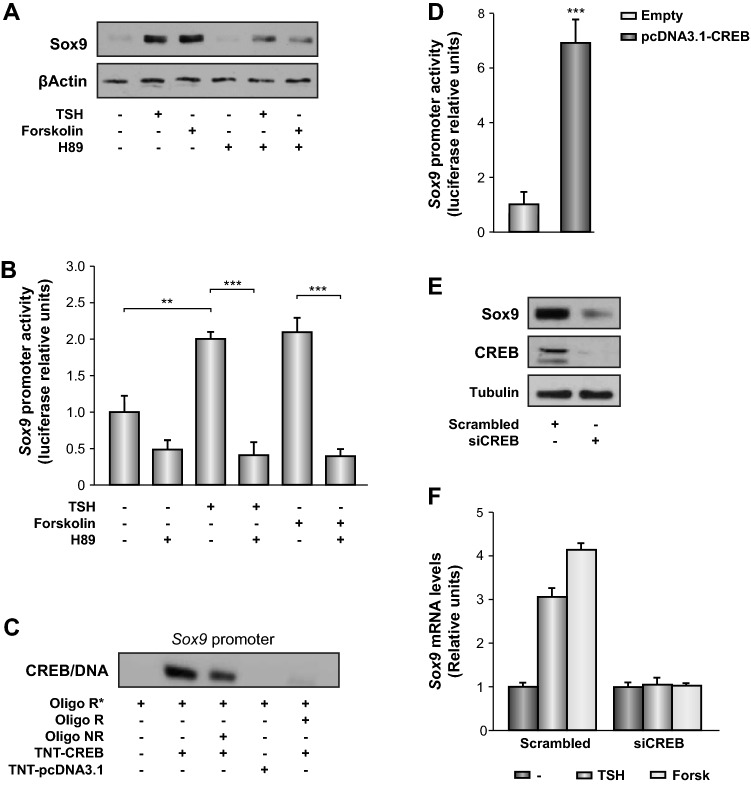
Analysis of the cAMP/CREB signaling pathway during thyrotropin (TSH)-mediated regulation of *Sox9* expression. (**A**) PCCl3 cells were maintained for 48 h in starvation medium (–) and then treated with different stimuli for 24 h. The H89 inhibitor was added 1.5 h before the treatment with TSH (1 nM) or forskolin (10 µM). Total protein extracts were analyzed by western blotting for the detection of Sox9. β-actin was used as a loading control. (**B**) PCCl3 cells were transfected with p*Sox9* and luciferase/*Renilla*-encoding pRL-CMV vectors. After transfection, cells were grown for 24 h in complete medium and then for 48 h in starvation medium (–) before treatment with TSH or forskolin for 24 h. The H89 inhibitor was added 1.5 h before the treatment, when necessary. Relative luciferase activity is represented as fold induction over the value in starved cells. (**C**) Electrophoretic mobility shift assays (EMSAs) were performed with a labeled cAMP-response element (CRE) sequence derived from the *Sox9* promoter. The labeled probe (R*) was incubated without protein or with 7 µg of CREB (TNT-CREB) recombinant protein. For competition, a 100-fold excess of the same related (R) or non-related (NR) cold oligonucleotide was used. (**D**) HeLa cells were transfected with p*Sox9 *and co-transfected with a CREB expression vector. Results represent fold induction with respect to the control. (E and F) PCCl3 cells were transfected with control (scrambled) or CREB (si*CREB*) small interfering RNAs (siRNAs). (**E**) Total protein extracts were obtained 24 h after transfection and analyzed by western blotting for the detection of Sox9 and CREB. β-actin was used as a loading control. (**F**) PCCl3 cells were maintained for 48 h in starvation medium (–) after transfection and then treated with TSH or forskolin for 16 h. *Sox9* mRNA expression levels were detected by RT-qPCR and normalized to those of β-actin. Values represent the mean ± standard error of the mean (SEM; n = 3). *P < 0.05; **P < 0.01; ***P < 0.001. Original blots/gels are presented in Supplementary Fig. 5.

The finding that *Sox9* expression is retained in the adult thyroid prompted us to study its function in follicular cell differentiation. We analyzed its expression in PCCl3 thyroid cells precultured in starvation medium and then treated with physiological doses of TSH. We found that hormone treatment markedly increased nuclear Sox9 protein levels visualized by confocal microscopy (Supplementary Fig. 1) and total protein determined by western blotting (Fig. 2A).

To test the involvement of the TSH-stimulated cAMP/PKA pathway, we treated PCCl3 cells with forskolin, an adenylate cyclase activator, or with the PKA inhibitor H89, prior to TSH/forskolin stimulation. We found that forskolin mimicked the effect of TSH on *Sox9 *expression whereas H89 inhibited both TSH- and forskolin-induced *Sox9* expression (Fig. 2A). Inhibitors of the PKC or MAPK pathway failed to abolish the TSH-mediated effect on *Sox9* expression (Supplementary Fig. 2A). The regulation of by TSH occurred at the transcriptional level, as TSH stimulated the activity of a transiently-transfected construct containing* Sox9* regulatory regions fused to the luciferase reporter gene^36^ (Fig. 2B). Altogether, these data suggest that TSH transcriptionally regulates *Sox9* expression via cAMP/PKA signaling.

Consistent with the involvement of the cAMP/PKA pathway in *Sox9* expression, in silico analysis of the *Sox9* gene regulatory region (~ 1 kb upstream from transcription initiation site) identified a putative cAMP response element (CRE) at position -202/-209 from the transcriptional start site (Supplementary Fig. 3A). We therefore studied the role of CREB, the major mediator of cAMP/PKA signaling, on *Sox9* transcription. Electrophoretic mobility shift assay (EMSA) revealed that recombinant CREB protein could specifically bind to the putative CRE (Fig. 2C) and was functional when co-transfected with a *Sox9-Luc* promoter construct (including the putative CRE site), as shown by a significant increase in luciferase activity (Fig. 2D).

We next silenced *CREB* gene expression in PCCl3 cells using siRNA, and this resulted in a marked decrease in Sox9 protein levels (Fig. 2E). Likewise, silencing *CREB *expression blunted TSH- and forskolin-induced *Sox9* expression (Fig. 2F). Overall, these results indicate that TSH/cAMP-dependent stimulation of *Sox9* expression is mediated, at least in part, by the CREB transcription factor.

### TGFβ inhibits the stimulating effect of TSH on *Sox9* expression

TGFβ signaling is operative in thyroid follicular cells and is known to be involved in the differentiation and pathophysiology of thyroid follicular cells^23,24^. Indeed, we previously demonstrated that TGFβ prevents TSH-dependent induction of thyroid differentiation gene expression^26,27^. Based on this evidence, we sought to determine the potential role of TGFβ in the regulation of *Sox9* expression.

**Figure 3:**
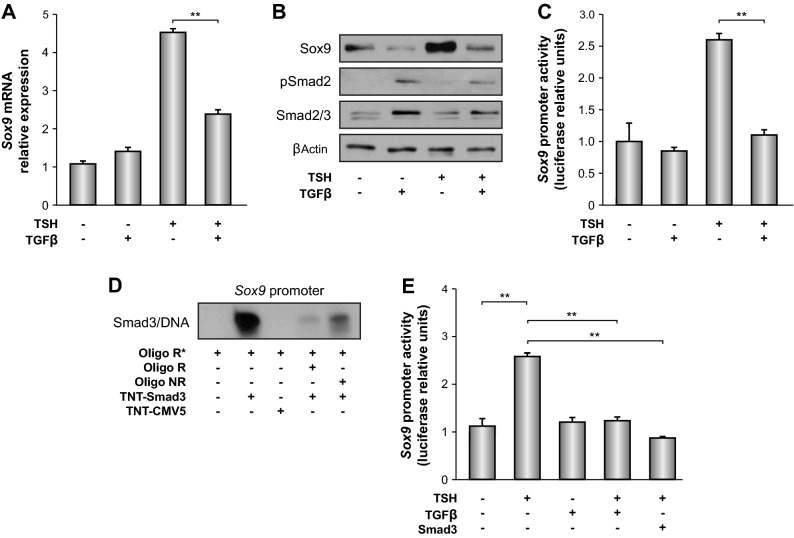
Role of Smad proteins in the inhibitory effect of TGFβ on TSH-induced *Sox9* expression. PCCl3 cells were maintained for 48 h in starvation medium (–) and then treated or not with TSH + TGFβ for (**A**) 16 h or (**B**) 24 h. *Sox9* mRNA expression levels were detected by RT-qPCR and normalized to those of *β-actin*. Total protein extracts were analyzed by western blotting for the detection of Sox9. β-actin was used as a loading control. pSmad2 was used as a control of Smad signaling activation. (**C**) PCCl3 cells were transfected with p*Sox9*-firefly luciferase and with *Renilla* luciferase-encoding pRL-CMV vectors. After transfection, cells were grown for 24 h in complete medium and then maintained 48 h in starvation medium before treatment with TSH or TSH + TGFβ for 24 h. Relative luciferase activity is represented as fold induction over the value of starved cells. (**D**) An electrophoretic mobility shift assay (EMSA) was performed with a labeled oligonucleotide corresponding to the Smad binding site identified in the *Sox9* promoter. The probe was incubated without protein or with 7 µg Smad3 (TNT-Smad3) recombinant protein, as indicated. For competition, a 100-fold excess of related (R) or non-related (NR) cold oligonucleotide was used. (**E**) *Sox9* promoter activity was analyzed in PCCl3 cells co-transfected with p*Sox9* and a *Smad3* expression vector and treated with TSH and TGFβ, as described in (**C**). Results show the fold induction of p*Sox9* with respect to the control (–). Values represent means ± SEM (n = 3). *ns* not significant; *P < 0.05; **P < 0.01; ***P < 0.001. Original blots/gels are presented in Supplementary Fig. 5.

We first treated starved PCCl3 cells with exogenous TSH and/or TGFβ. We found that while TGFβ alone had no effect on *Sox9 *expression, it blocked the TSH-dependent increase of *Sox9* mRNA (Fig. 3A) and protein (Fig. 3B) levels. These effects occurred at the transcriptional level, as TGFβ also inhibited the TSH-dependent induction of *Sox9* promoter activity in transient transfections assays (Fig. 3C). TGFβ signaling is critically dependent on Smad signaling proteins, and we identified a putative Smad-binding site within the *Sox9* promoter (Supplementary Fig. 3A). EMSA showed that recombinant Smad3 bound specifically to an oligonucleotide containing the Smad-binding site located at -25/-17 from the transcription initiation site of the *Sox9* gene (Fig. 3D). Furthermore, *Smad3* overexpression in PCCl3 cells mimicked the inhibitory effect of TGFβ on the TSH-dependent activation of the *Sox9* promoter (Fig. 3E). Taken together, these data demonstrate that TGFβ inhibition of TSH-dependent stimulation of *Sox9* promoter activity occurs via Smad proteins.

### Sox9 is involved in the transcriptional network that regulates thyroid differentiation

Thyroid differentiation is regulated by the simultaneous expression of the thyroid transcription factors Nkx2.1, Foxe1 and Pax8^37^. In silico analysis identified putative binding sites for all three transcription factors within the ~ 1-kb *Sox9* regulatory region (Supplementary Fig. 3A). We therefore postulated that Sox9 might play a role in the differentiation of thyroid follicular cells.

**Figure 4:**
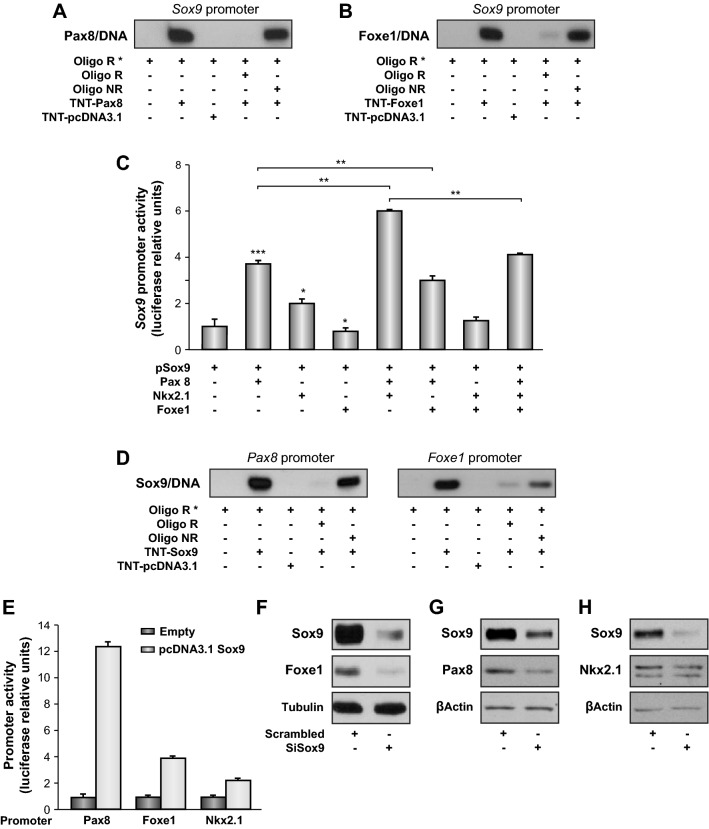
Involvement of Sox9 in the thyroid transcriptional regulatory network. (**A** and **B**) Electrophoretic mobility shift assays (EMSAs) were performed with labeled oligonucleotides derived from the *Sox9* regulatory regions. The probes were incubated without protein or with 7 µg (**A**) Pax8 (TNT-Pax8) or (**B**) Foxe1 (TNT-Foxe1) recombinant proteins. For competition, a 100-fold excess of related (R) or nonrelated (NR) cold oligonucleotide was used. (**C**) HeLa cells were co-transfected with p*Sox9* and the indicated expression vectors (*Pax8, Nkx2.1*, or *Foxe1*). Results show the fold induction of p*Sox9* with respect to the control (–). (**D**) EMSAs were performed with labeled oligonucleotides derived from the *Pax8* and *Foxe1* promoters. The probes were incubated without protein or with 7 µg Sox9 (TNT-Sox9) recombinant protein. For competition, a 100-fold excess of related (R) or non-related (NR) cold oligonucleotide was used. (**E**) HeLa cells were co-transfected with p*Foxe1*, p*Nkx2.1* or p*Pax8* and the *Sox9* expression vector. Results show the fold induction of *Foxe1, Nkx2.1* and *Pax8* promoters with respect to the control (–). (F, G, H) PCCl3 cells were transfected with control (scrambled) or Sox9 (si*Sox9*) siRNAs. Total protein extracts were obtained 24 h after transfection and analysed by western blotting for Sox9 and Foxe1 (**F**), Sox9 and Pax8 (**G**) and Sox9 and Nkx2.1 (**G**). Tubulin or β-actin was used as a loading control. Values represent means ± SEM (n = 3). *P < 0.05; **P < 0.01; ***P < 0.001. Original blots/gels are presented in Supplementary Fig. 5.

As a first approach, we analyzed the ability of the thyroid transcription factors to bind to the *Sox9* promoter elements. EMSAs were performed using oligonucleotides containing the putative binding sites for Nkx2.1 (at -280/-271 and at -7/-15), Pax8 (at + 35/ + 43) and Foxe1 (at -652/-647) identified in silico and using proteins translated in vitro to generate recombinant transcription factors. Results showed a clear and specific band shift for Pax8 and Foxe1 (Fig. 4A,B), suggesting that both thyroid transcription factors can bind to the *Sox9* promoter. While Nkx2.1 was also able to bind to its putative -7/-15 site, the binding was not specific since the non-related oligonucleotide competed as efficiently as the related oligonucleotide (Supplementary Fig. 4A). A similar lack of specificity for Nkx2.1 binding was observed when the putative -280/-271 site was assessed (data not shown). We next questioned whether the specific binding of Pax8 and Foxe1 was transcriptionally functional, and would result in the regulation of *Sox9* promoter activity. To do this, we co-transfected HeLa cells with the *Sox9*-*Luc* construct (p*Sox9* in Fig. 4C) and expression vectors for *Pax8*, *Nkx2.1*, and *Foxe1* alone or in all possible combinations. Results revealed that Pax8 had the greatest and most significant positive effect on *Sox9* reporter activity (Fig. 4C). Despite the non-specific binding determined by EMSA, Nkx2.1 modestly increased *Sox9* reporter activity (Fig. 4C), reflecting its weak binding to the *Sox9* promoter (Supplementary Fig. 4A). Notably, Nkx2.1 cooperated with Pax8 to further stimulate *Sox9* reporter activity, having an additive effect (Fig. 4C). By contrast, Foxe1 overexpression, alone or in combination with Nkx2.1 and Pax8, decreased *Sox9* reporter activity, suggesting a repressive effect on *Sox9* transcription.

Our findings highlight Sox9 as a transcription factor that is finely tuned in the thyroid, suggesting it could be part of the transcriptional network of genes regulating thyroid differentiation. We therefore examined whether Sox9 controls the expression of the thyroid transcription factors *Nkx2.1*, *Pax8* and *Foxe1*. Putative binding sites for Sox9 were identified in the promoters of all three genes by in silico analyses. EMSA with recombinant Sox9 indicated that it bound strongly and specifically to putative sites located -389 and -2268 bp from the transcription initiation site of *Pax8* and *Foxe1*, respectively (Fig. 4 D). Weaker and non-specific binding of Sox9 to the -274 site of the *Nkx2.1* regulatory region was also observed (Supplementary Fig. 4B). Binding of Sox9 was functional, as co-transfection of the *Sox9 *expression vector in Hela cells stimulated the activity of all three promoters, albeit to a different extent (Fig. 4E). The maximum activity (12-fold) was elicited on the *Pax8* promoter. Sox9 stimulated the *Foxe1* and *Nkx2.1* promoters four- and two-fold, respectively. These data confirm that Sox9 is directly involved in the transcriptional regulation of the three thyroid-specific transcription factors, with a major effect on *Pax8* expression. To validate these finding we used a *Sox9-*specific siRNA to silence its expression in PCCl3 cells. Western blotting of *Sox9*-silenced PCCl3 cells caused a concomitant decrease in the steady-state levels of Foxe1 and Pax8 (Fig. 4F,G), but not of Nkx2.1 (Fig. 4H).

Overall, these data suggest that Sox9 might be part of the network of transcription factors that control thyroid cell differentiation as revealed by the observed in vitro inter-relationship between Pax8 and Sox9. Moreover, our findings on the extrinsic regulation of *Sox9* expression by TSH and TGFβ support the potential importance of this transcription factor in thyroid follicular cells, not only during development but also in adulthood.

## Discussion

Thyroid differentiation begins during embryonic development and the differentiated state persists throughout adult life to maintain the production of T3 and T4 by the thyroid gland. Our knowledge of the mechanisms that govern this process has advanced considerably in recent years, resulting in the identification of many new actors^2,4^. Among these is the transcription factor Sox9, which is involved in thyroid branching morphogenesis, a fundamental mechanism in the embryonic development of various organs^7^. In adults, Sox9 expression appears to be exclusive to follicular cells, as it is undetectable in C cells and connective tissue cells^7^. Here we show that the expression of *Sox9,* observed in precursor cells of the thyroid primordium, persists in adult follicular cells with an evident nuclear localization. The finding that *Sox9* expression is more robust in adult thyroid follicle cells than in thyroid embryonic cells suggests that it plays a role in the maintenance of thyroid follicle cell differentiation. Likewise, the discovery that TSH finely regulates *Sox9* might indicate a role for this transcription factor in chronically challenging thyroid states, such as hypo- and hyper-thyroidism. In addition to TGFβ, other molecular signals controlling thyroid differentiation also control* Sox9* expression. This resembles the embryonic situation where Fgf10, through its receptor Fgfr2b, regulates *Sox9* expression during thyroid branching^7^. Sox9 is therefore likely at the center of an important regulatory network of signals that govern thyroid differentiation both in embryos and in adults. This potentially important role for Sox9 is reinforced by the finding that its promoter is conserved between rodents and humans (Supplementary Fig. 3B).

The finding that forskolin mimics the action of TSH on *Sox9* together with the positive action of PKA inhibition and the lack of effect of both PKC and the MAPK inhibitors over TSH, convincingly demonstrates that the effects of TSH on *Sox9* are mediated by cAMP/PKA. We would note that the present study was performed using physiological doses of TSH that do not activate the aforementioned pathways^22,38^ (Supplementary Fig. 2B). Finally, the influence of TSH/cAMP/PKA occurs at the transcriptional level through the binding of CREB to a CRE site in the *Sox9* promoter. Silencing of *CREB* expression dramatically reduced the abundance of *Sox9* mRNA and protein, and silenced cells lost their responsiveness to TSH and forskolin, overall demonstrating that the CREB transcription factor is essential for the regulation of *Sox9* by TSH via cAMP.

Similar to other thyroid differentiation genes^27,31^, TGFβ inhibited the inductive effect of TSH on *Sox9* expression, a transcriptional mechanism mediated by Smad proteins. Indeed, overexpression of Smad3 inhibited TSH-induced *Sox9* promoter activity independently of TGFβ addition. The finding that *Sox9* is negatively regulated by TGFβ confirms the existence of a homeostatic balance between the TSH/cAMP and TGFβ/Smads pathways to control thyroid follicular cell differentiation. Indeed, in a recent study designed to improve in vitro protocols for functional thyroid organoid cultures, the authors, guided by single-cell RNA sequencing, found that the addition of cAMP activators and TGFβ inhibitors to the differentiation medium improved thyroid maturation and functional activity^39^. Thus, the positive action of cAMP and the blocking negative action of TGFβ are likely crucial factors for thyroid maturation, and perhaps for Sox9-induced branching.

Our results suggest that Sox9 is involved in the hormonal and transcriptional regulatory network that controls the maintenance of thyroid differentiation. As previously demonstrated^32^, thyroid transcription factors are linked in an integrated regulatory system. In this context, we identified DNA binding sites for the thyroid transcription factors Nkx2.1, Foxe1 and Pax8 within the *Sox9* promoter, and all appeared to bind, albeit to a different extent. A possible explanation for the different binding ability of each factor might be the absence of coactivators, as EMSA studies are in vitro experiments that analyze transcription factors/DNA interactions. Functional experiments would suggest that this is the case, as the three transcription factors increased the activity of *Sox9* promoter individually in transient transfection experiments, but performed better in combination (Fig. 4C). Accordingly, Pax8 had a greater stimulatory effect than Nkx2.1 on *Sox9 *expression, but simultaneous binding of both factors were additive. The functional cooperation between Nkx2.1 and Pax8 has been previously described for other thyroid differentiation genes such as *Tg,* and this effect is mediated respectively by their N- and C-terminal domains^40^. The superior performance of Pax8 in the regulation of *Sox9* artificial reporter construct confirms its fundamental role in thyroid differentiation^41^. Interestingly, Foxe1 elicited a repressive effect on *Sox9* promoter activity, confirming a previous report^42^. Moreover, Foxe1 also repressed the inducing activity of Nkx2.1 and Pax8 alone or together. This observation supports the study of Perrone et al. (2000), which showed that Foxe1 inhibits the activity of Nkx2.1 and Pax8 only on certain promoters^42^. The repressor activity of Foxe1 is borne by its C-terminal region, which contains an alanine-rich domain. Foxl2, which is another forkhead transcription factor, has been shown to inhibit ovarian expression of *Sox9* by synergistic binding with the estrogen receptor to an enhancer present in the regulatory region of *Sox9*. The absence of Foxl2 leads to enhanced *Sox9* expression and ovary cell transdifferentiation^43^. Future studies should examine the mechanisms through which Foxe1 inhibits *Sox9* promoter activity, and their relationship in early development. This would provide information on whether Foxe1, through its inhibitory actions, functions as a temporary modulator of Sox9 activity during thyroid follicular cell differentiation.

The involvement of Sox9 in thyroid follicular cell transcriptional regulation networks was also supported by the finding of Sox9-binding sites in *Nkx2.1*, *Foxe1* and *Pax8* promoters, which appeared to be functional. It is currently accepted that Sox9 activity depends not only on the consensus binding sequence in target promoters, but also on its interaction with other transcription factors. For example, Sox9 forms homo- and hetero-dimers with other transcription factors such as the Sonic Hedgehog mediator Gli^44^ or CREB^45^. Thus, it will be important in the future to study whether homodimerization of Sox9 and its heterodimerization with other Sox factors or proteins such as CREB or thyroid transcription factors, differentially controls the expression of thyroid differentiation genes.

The present study provides new data on the reciprocal regulation of *Pax8*, *Foxe1* and *Sox9* expression and opens new avenues to better understand the complex network controlling thyroid differentiation. We speculate that Sox9 might play an important role in the regulation of thyroid differentiation genes, such as *Tg* and *NIS*. Our preliminary results point in this direction and future work will be aimed at understanding these mechanisms of regulation. Along this line, a crucial yet poorly understood mechanism is thyroid folliculogenesis. Based on our previous results demonstrating a role for Pax8 in thyroid folliculogenesis^46^ and our new data describing the regulation of *Sox9* by Pax8, we believe that its role should be studied in 3D follicle formation assays, which better mimic the in vivo environment.

## Materials and methods

### Bioinformatic analysis

We utilized two bioinformatic tools to search for transcription factor binding sites in human, mouse and rat gene promoters: TRANSFAC (www.gene-regulation.com/pub/databases.html) and the ECR Browser (www.ecrbrowser.dcode.org). We used the NCBI/BLAST server (www.blast.ncbi.nlm.nih.gov) to compare different species promoter sequences from the NCBI/Gene database.

### Animals

The experimentation in mice was performed in compliance with the European Community Law (86/609/EEC). Studies in mice embryos were performed according to the guidelines of laboratory animal care of the University Animal Welfare Committee, Université Catholique de Louvain; the corresponding experiments in adult mice followed the guidelines of the Spanish law (R.D. 1201/2005), with the approval of the ethics committee of the Consejo Superior de Investigaciones Científicas (CSIC, Spain).

### Cell cultures

PCCl3 rat thyroid follicular cells are an extensively used thyroid model system, as they express thyroid-specific genes^47^. Cells were cultured in Coon’s modified Ham’s F-12 medium supplemented with 5% donor calf serum (Life Technologies, Inc., Gaithersburg, MD). The following 6-hormone mixture, 1 nM TSH, 10 µg/ml insulin, 10 ng/ml somatostatin, 5 µg/ml transferrin, 10 nM hydrocortisone, and 10 ng/ml glycil-L-histidyl-L-lysine acetate, as well as H89, forskolin and PDBU were purchased from Sigma-Aldrich Chemical Co. (St. Louis, MO). The effect of hormones and growth factors was studied after starving near confluent cells for TSH and insulin in the presence of 0.1% donor calf serum (starvation medium) for 2 days. Subsequently, the following agents were added at the final concentrations: TSH (1 nM), forskolin (10 µM), TGFβ (10 ng/ml), phorbol 12,13-dibutyrate (PDBU, 500 nM) and epidermal growth factor (EGF (10 ng/µl). The PKA inhibitor H89 (10 µM)^48^, the PKC inhibitor GF1 (3.5 µM)^49^ and the MEK1/2 inhibitor U0126 (10 µM)^50^ were added 90, 30 and 60 min, respectively, before treatment with the ligands. HeLa cells were cultured in Dulbecco’s modified Eagle’s medium (DMEM) supplemented with 10% fetal bovine serum, glutamine, antibiotics and sodium pyruvate. TGFβ was obtained from Peprotech (Rocky Hill, NJ), EGF from R&D Systems (Minneapolis, MN) and GF1 and U0126 from Calbiochem (San Diego, CA).

### RT-qPCR

Total RNA was extracted using the TRIzol reagent (Thermo Fisher Scientific, Waltham, MA) and 2 µg of RNA were used to obtain cDNA through a reverse transcription reaction (MMLV; Promega, Madison, WI). Quantitative RT-PCR was performed using specific primers (see Supplementary Table 1) using the KAPA Sybr Fast qPCR Master Mix (Merck KGaA, Darmstadt, Germany) for 40 cycles. The specificity of the reactions was determined by melting curve analysis. The number of cycles needed to reach the threshold level (Ct) for each sample was used to calculate the amount of each PCR product with the 2^−ΔΔCt^ method^51^. Relative levels of the PCR products were expressed as a function of *β-actin*. RT-qPCR was performed on a Mx3000P QPCR platform (Agilent Technologies).

### Western blotting

Total proteins were extracted and prepared as described^52^. Equal amounts of protein (20–30 µg) were separated by SDS-PAGE, transferred to nitrocellulose membranes, blocked in PBS-T buffer (PBS + 0.1% Tween 20 pH 7.5) containing 5% nonfat dry milk, and incubated overnight at 4ºC with the primary antibodies (described in Supplementary Table 2). After washing three times with PBS-T buffer, the membranes were incubated with the appropriate species-specific-HRP-conjugated secondary antibody for one hour. After another washing step, immunoreactive bands were detected with the Luminol Western blot detection reagent (Thermo Fisher Scientific).

### Transient transfection

200,000 cells were seeded in 6-well plates and transiently transfected with calcium chloride, after 24 hours^53^. *Sox9*^36^*, Nkx2.1*^54^, *Pax8*^55^ and *Foxe1*^27^ promoters fused to the luciferase reporter gene were transiently transfected with the following expression vectors: h*CREB*^56^, h*SMAD3/4*^57^, h*PAX8*^58^, r*Foxe1*^59^, r*Nkx2.1*^60^ and h*SOX9*^61^ or the corresponding empty vector. Transfections were performed with 1.5 µg/well of the promoter construct and 1.5 µg/well of the expression vector. To check the transfection efficiency, 0.1 µg of the CMV-*Renilla* promoter construct was added to each well. After 48 h, cells were harvested, lysed and analyzed using the luciferase reporter dual assay system (Promega) to measure luciferase and *Renilla* expression. Promoter activity was determined as the ratio of luciferase and *Renilla* levels and is represented as relative luciferase activity.

### Inmunohistochemistry

Paraffin-embedded thyroid sections were prepared for immunohistochemistry using the EnVision + System-HRP (DAB) Kit (Dako, Carpinterio, CA). Samples were deparaffined, rehydrated and treated with citrate buffer (0.01 M pH 6) for antigen retrieval. Endogenous peroxidase activity was inhibited with hydrogen peroxide (3%) and slides were incubated with the primary antibody (Supplementary Table 2) for one hour. After washing, sections were incubated with an HRP-conjugated secondary antibody for one hour and a deaminobenzine (DAB) solution was used for staining and detection of the antibody. Subsequently, sections were stained with hematoxylin, dehydrated and mounted on coverslips with DPX. Finally, the stained sections were examined using a Nikon 90i fluorescence microscope (Nikon, Japan).

### Inmunofluorescence

Embryonic tissues were obtained from mice at E9.5, E10.5 and E11.5 and were processed as in Villacorte, 2016^62^. Antigen–antibody complexes were visualized using Alexa 488-, 568- or 647-conjugated secondary antibodies (Invitrogen). Finally, nuclei were stained with Hoechst fluorescent dye (Hoechst 33,258; Sigma) and sections examined with a spinning disk confocal microscope using a Plan Apochromat 100x/1.4 Oil DIC objective (Cell Observer Spinning Disk; Zeiss).

Transgenic mice expressing *GFP *under the control of *Sox9* regulatory elements^34,35^ were also used for immunofluorescence studies. Tissue samples were extracted at E16.5 or from adult mice, embedded in gelatin and processed as described above. Tissues were also analyzed using a spinning disk confocal microscope (Cell Observer Spinning Disk; Zeiss).

When using cell lines for immunofluorescence, cells were seeded on coverslips and fixed with 4% paraformaldehyde before blocking and incubation with antibodies. Samples were examined with a *Leica TCS SP5* confocal microscope (Leica Systems GmbH, Watzlar, Germany).

### Gene silencing

PCCl3 cells were silenced using an ON-TARGET plus SMARTpool small interfering RNA (siRNA) against *Sox9* or *CREB* (Dharmacon, GE Healthcare, Lafayette, CO). Cells were harvested 24 and 48 h post-transfection, and total RNA and protein were extracted as described. A non-targeting siRNA (scrambled siRNA) was used as a negative control.

### Electrophoretic mobility shift assays

Oligonucleotides corresponding to the binding sites of CREB, Smad3, Pax8, Foxe1 and Nkx2.1 in the *Sox9* promoter and of Sox9 in the *Pax8, Foxe1* and *Nkx2.1* promoters (see Supplementary Table 3) were labeled with 25 µCi of ɣ^32^P-ATP using T4 polynucleotide kinase (Promega) and were purified on QuickSpin G-25 Sephadex columns (Roche Life Sciences, Mannheim, Germany). Recombinant proteins were produced with the in vitro transcription-translation (TNT) kit (Promega) and incubated with the labeled probes. For electrophoretic mobility shift assay (EMSA), binding reactions were performed in a buffer containing 40 mM Hepes pH 7.9, 75 mM KCl, 0.2 mM EDTA, 0.5 mM dithiothretiol, 150 ng/µl poly (dI-dC) and 5% Ficoll at room temperature for 30 min. Samples were electrophoresed on 5% polyacrylamide geld in 0.5 × Tris–borate EDTA. To evaluate the specificity of the DNA–protein interactions, labeled probes were incubated with an excess (50-fold) of the same (related) or different (unrelated) unlabeled oligonucleotides.

### Statistical analysis

Results are represented as fold induction ± standard error of the mean from at least three different experiments. Student’s two-tailed t-test was used to assess the differences between measurements. A *P*-value of ˂0.05 was considered statistically significant.

## Supplementary Information

Supplementary Information 1.

Supplementary Information 2.

Supplementary Information 3.

Supplementary Information 4.

Supplementary Information 5.

Supplementary Information 6.
