# Supplementary material for: Sox9 is involved in the thyroid differentiation program and is regulated by crosstalk between TSH, TGFβ and thyroid transcription factors
Source: Sci Rep. 2022 Feb 9;12:2144. doi: 10.1038/s41598-022-06004-1 (PMC8828901; doi:10.1038/s41598-022-06004-1)
Supplement: Supplementary file 1 — Supplementary Information 1. [file 41598_2022_6004_MOESM1_ESM.pdf]

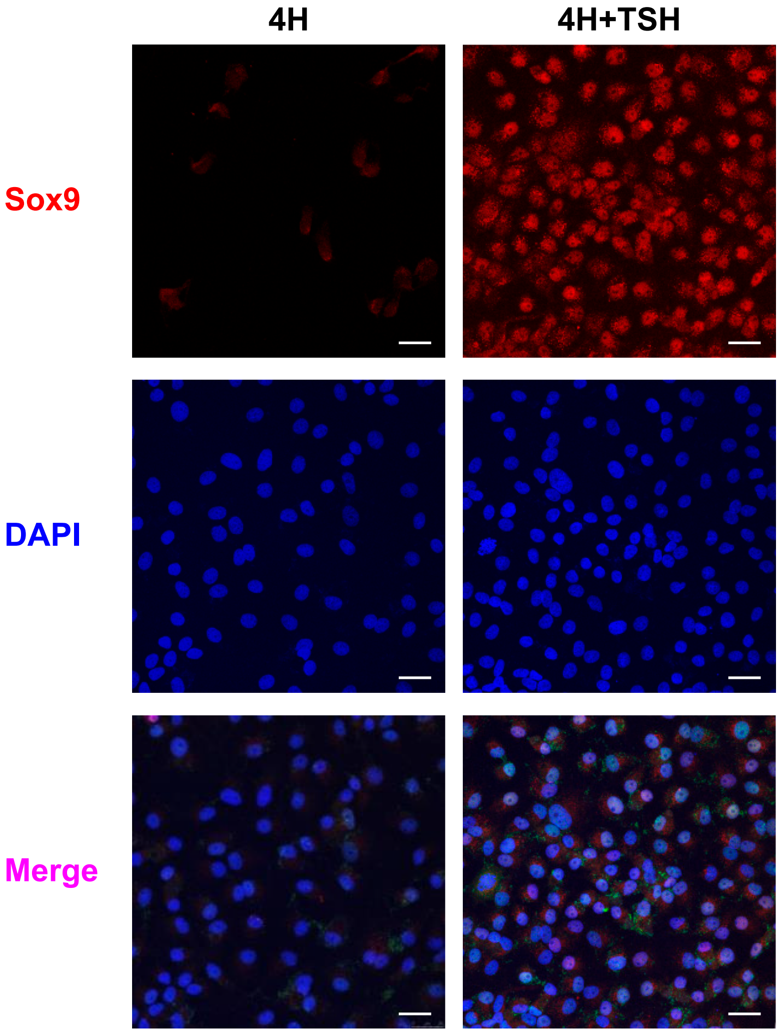

Supplementary Figure 1. Inmunofluorescence of Sox9. PCCl3 cells were cultured for 48 hours in starvation medium (–) and then treated with TSH (1 nM) for 24 hours. Nuclei were labelled with DAPI.
