# Supplementary material for: Sox9 is involved in the thyroid differentiation program and is regulated by crosstalk between TSH, TGFβ and thyroid transcription factors
Source: Sci Rep. 2022 Feb 9;12:2144. doi: 10.1038/s41598-022-06004-1 (PMC8828901; doi:10.1038/s41598-022-06004-1)
Supplement: Supplementary file 2 — Supplementary Information 2. [file 41598_2022_6004_MOESM2_ESM.pdf]

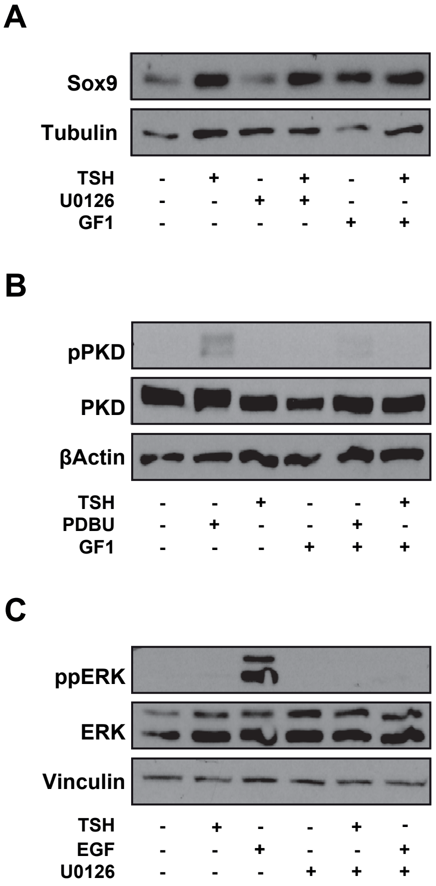

Supplementary Figure 2. Analysis of PKC and MAPK signaling pathways during thyrotropin (TSH)-mediated regulation of *Sox9*. (A) PCCl3 cells were maintained for 48 hours in starvation medium (–) and then treated with 1 nM TSH for 48 hours. The GF1 and U0126 inhibitors were added 30 and 60 minutes, respectively, before the treatment. Total protein extracts were analyzed by western blotting for the detection of Sox9. Tubulin was used as a loading control. (B) PCCl3 cells were maintained for 48 hours in starvation medium (–) and then treated with different stimuli for 5 minutes. 500 nM PDBU (phorbol 12,13-dibutyrate) and 10ng/ml EGF (epidermal growth factor) were used to activate PKC and MAPK pathways, respectively. The GF1 and U0126 inhibitors were used as in panel A. Total protein extracts were analyzed by western blotting for the detection of pPKD, and PKD (left panel) and ppERK and ERK (right panel). β-Actin and vinculin were used as loading controls.
