# Supplementary material for: Sox9 is involved in the thyroid differentiation program and is regulated by crosstalk between TSH, TGFβ and thyroid transcription factors
Source: Sci Rep. 2022 Feb 9;12:2144. doi: 10.1038/s41598-022-06004-1 (PMC8828901; doi:10.1038/s41598-022-06004-1)
Supplement: Supplementary file 3 — Supplementary Information 3. [file 41598_2022_6004_MOESM3_ESM.pdf]

A

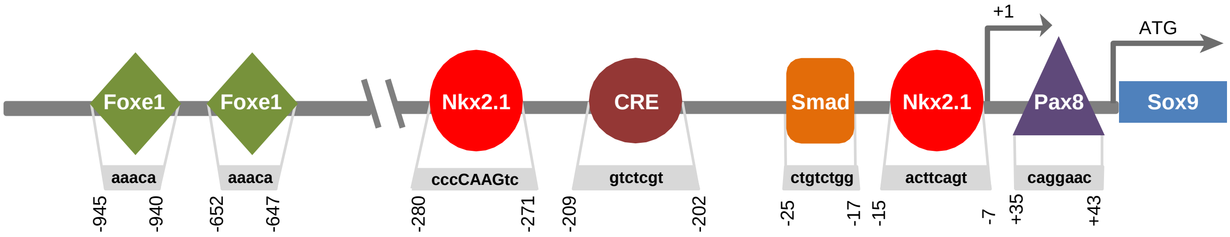

B

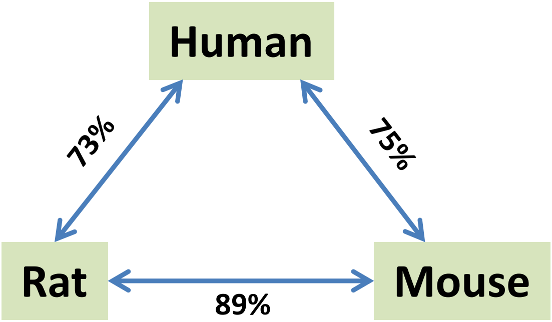

Supplementary Figure 3. (A) Schematic representation of the promoter region of the rat *Sox9* gene. The geometric drawing shows the binding sites, identified in silico, of the different transcription factors studied. The putative consensus sequences identified are marked below each transcription factor binding site, together with their relative position from the transcriptional start site (+1). (B) Homology (percentage) between the regulatory regions (1300 base pairs upstream from the translation start point) of *Sox9* in human, mouse and rat.
