# Supplementary material for: Sox9 is involved in the thyroid differentiation program and is regulated by crosstalk between TSH, TGFβ and thyroid transcription factors
Source: Sci Rep. 2022 Feb 9;12:2144. doi: 10.1038/s41598-022-06004-1 (PMC8828901; doi:10.1038/s41598-022-06004-1)
Supplement: Supplementary file 4 — Supplementary Information 4. [file 41598_2022_6004_MOESM4_ESM.pdf]

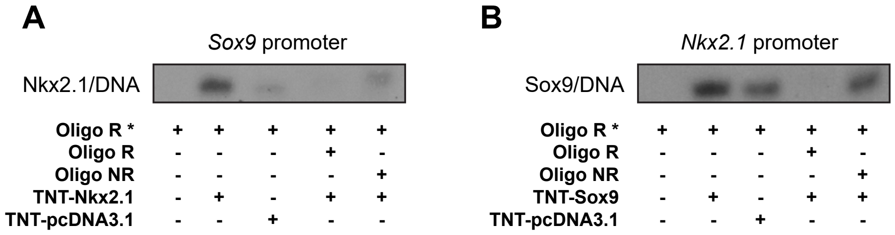

Supplementary Figure 4. (A) Electrophoretic mobility shift assay (EMSA) was performed with a labelled Nkx2-1 binding sequence within the *Sox9* promoter. The labelled probe (R*) was incubated without protein or with 7 μg of Nkx2-1 (TNT-Nkx2-1) recombinant protein. For competition, a 100-fold excess of the same related (R) or non-related (NR) cold oligonucleotide was used. (B) Electrophoretic mobility shift assay (EMSA) was performed with a labelled Sox9 binding sequence within the *Nkx2-1* promoter. The labelled probe (R*) was incubated without protein or with 7 μg of Sox9 (TNT-Sox9) recombinant protein. For competition, a 100-fold excess of the same related (R) or non-related (NR) cold oligonucleotide was used.
