# Supplementary material for: Sox9 is involved in the thyroid differentiation program and is regulated by crosstalk between TSH, TGFβ and thyroid transcription factors
Source: Sci Rep. 2022 Feb 9;12:2144. doi: 10.1038/s41598-022-06004-1 (PMC8828901; doi:10.1038/s41598-022-06004-1)
Supplement: Supplementary file 5 — Supplementary Information 5. [file 41598_2022_6004_MOESM5_ESM.pdf]

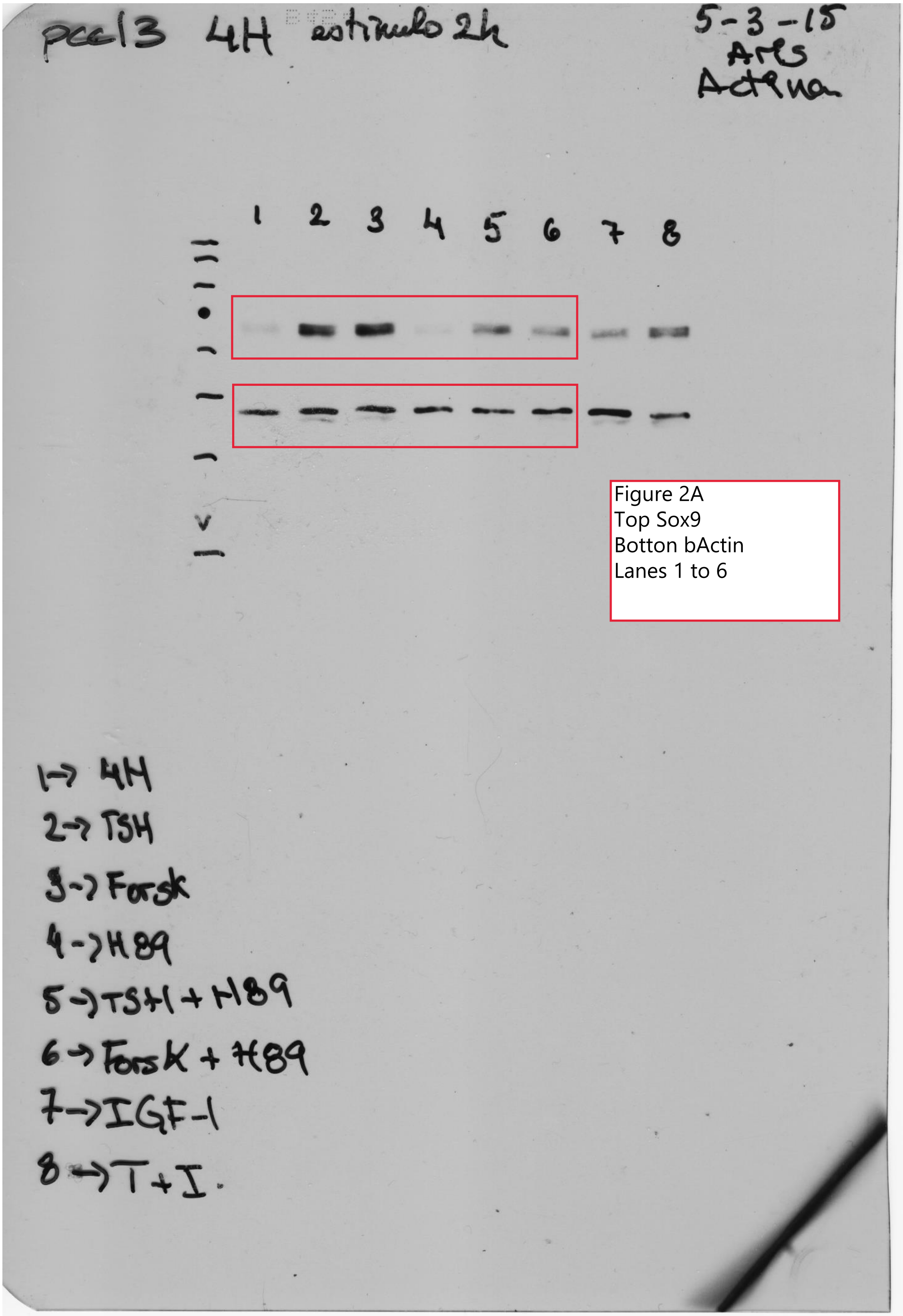

Figure 2A
Top Sox9
Botton bActin
Lanes 1 to 6

Fig 2C EMSA CREB/
Sox9

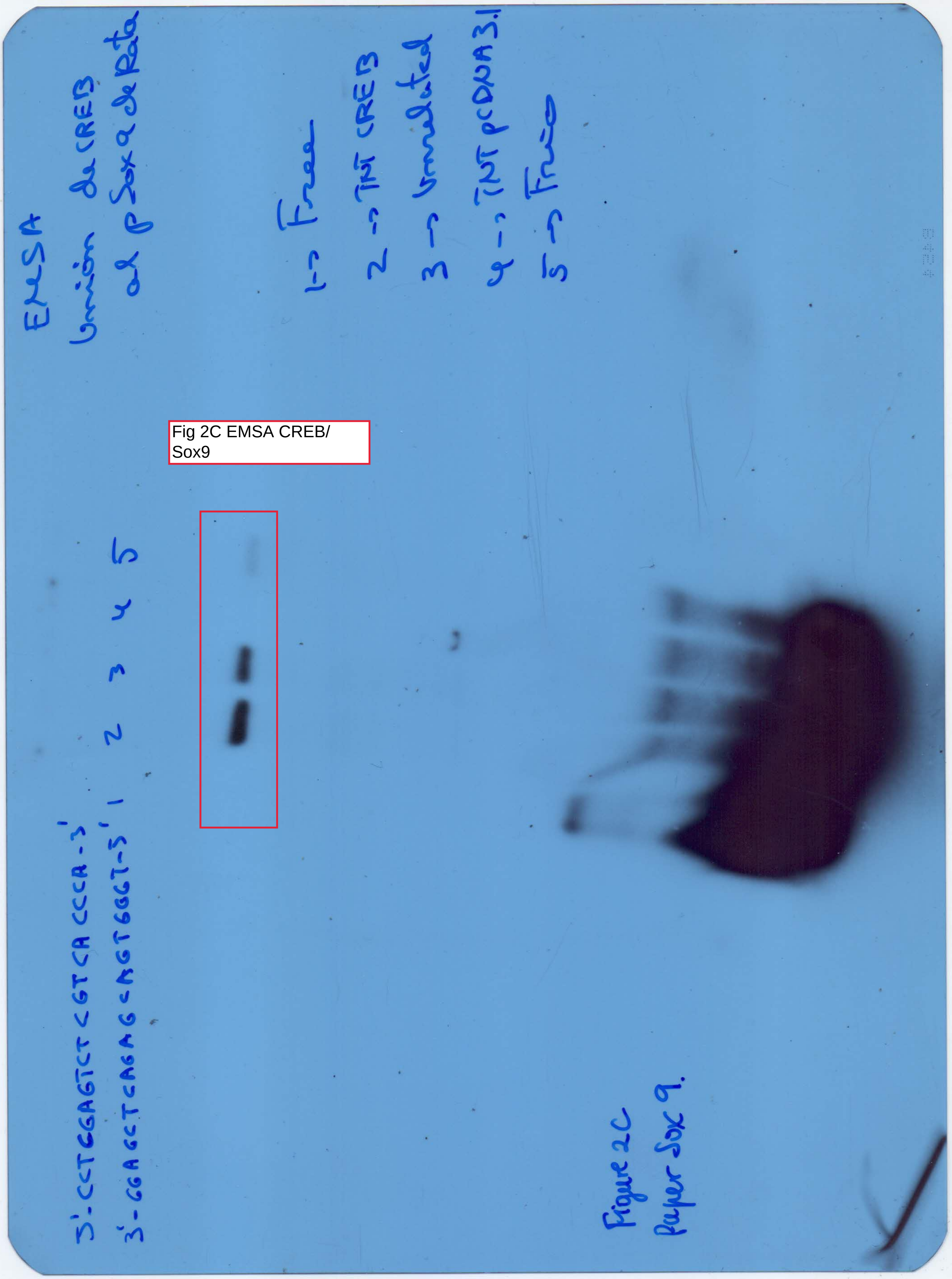

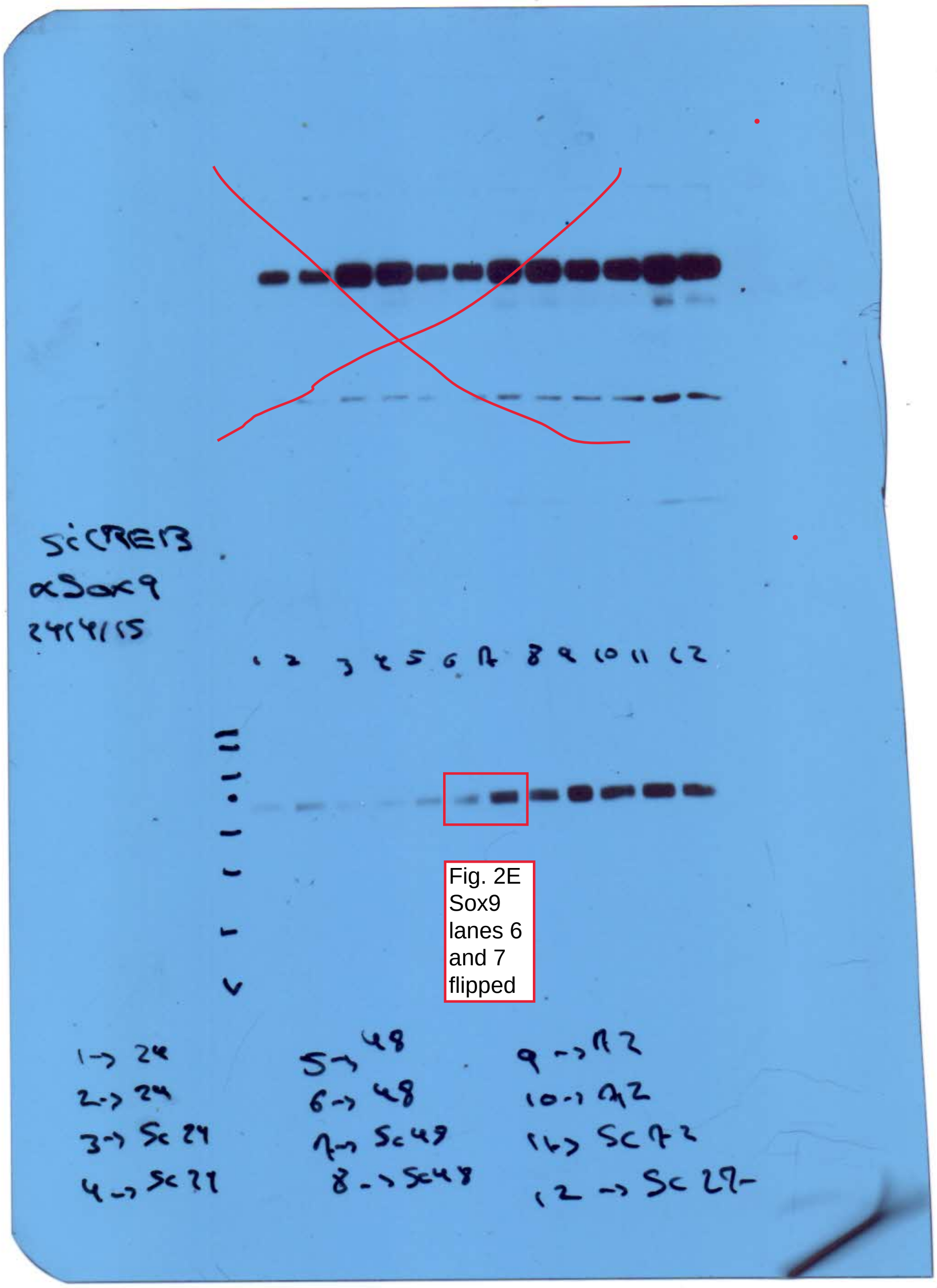

Fig. 2E
Sox9
lanes 6
and 7
flipped

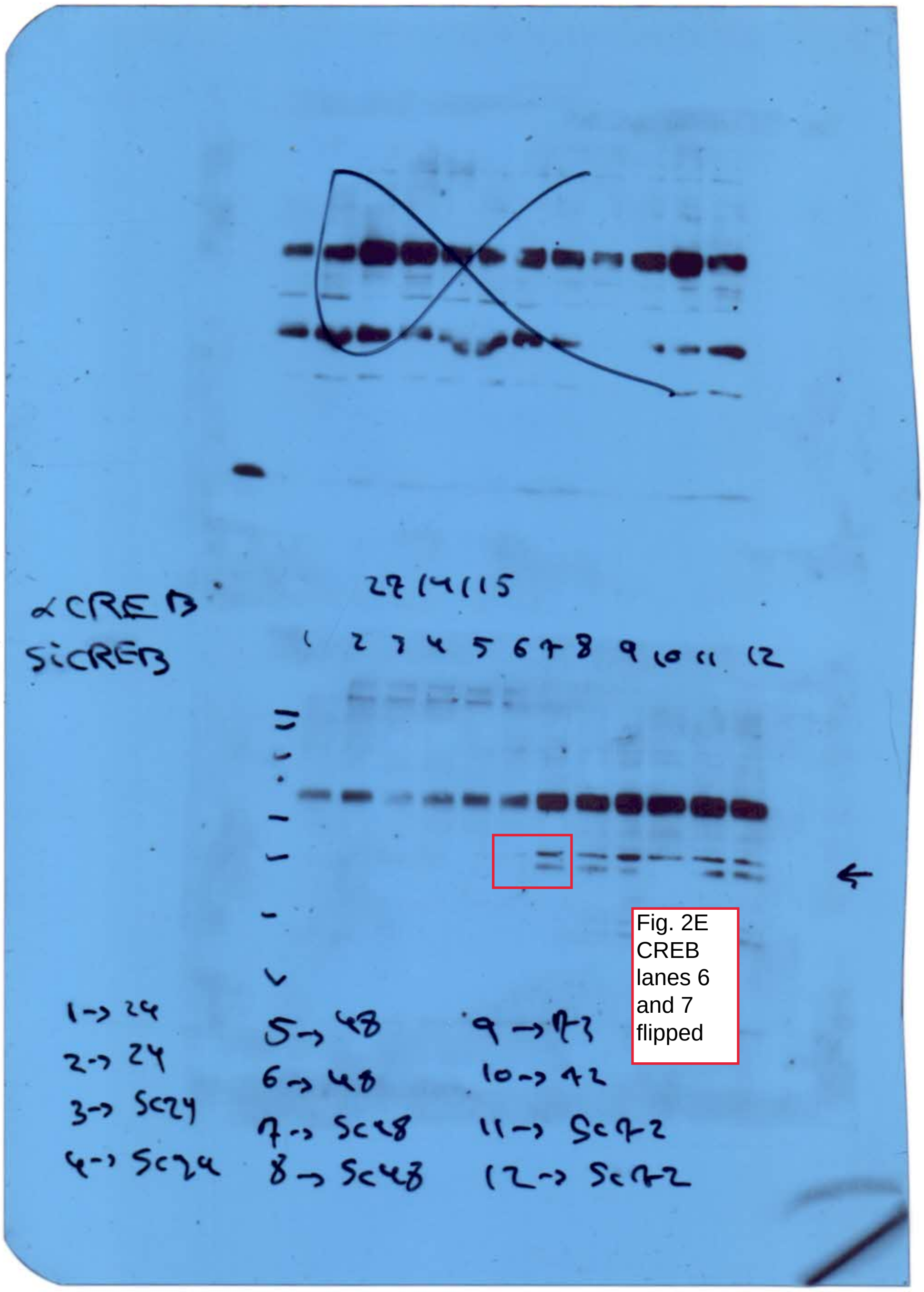
αCREB
SiCREB
27/4/15
1 2 3 4 5 6 7 8 9 10 11 12
1 -> 24
2 -> 24
3 -> Sc24
4 -> Sc24
5 -> 48
6 -> 48
7 -> Sc48
8 -> Sc48
9 -> 72
10 -> 72
11 -> Sc72
12 -> Sc72
Fig. 2E CREB lanes 6 and 7 flipped

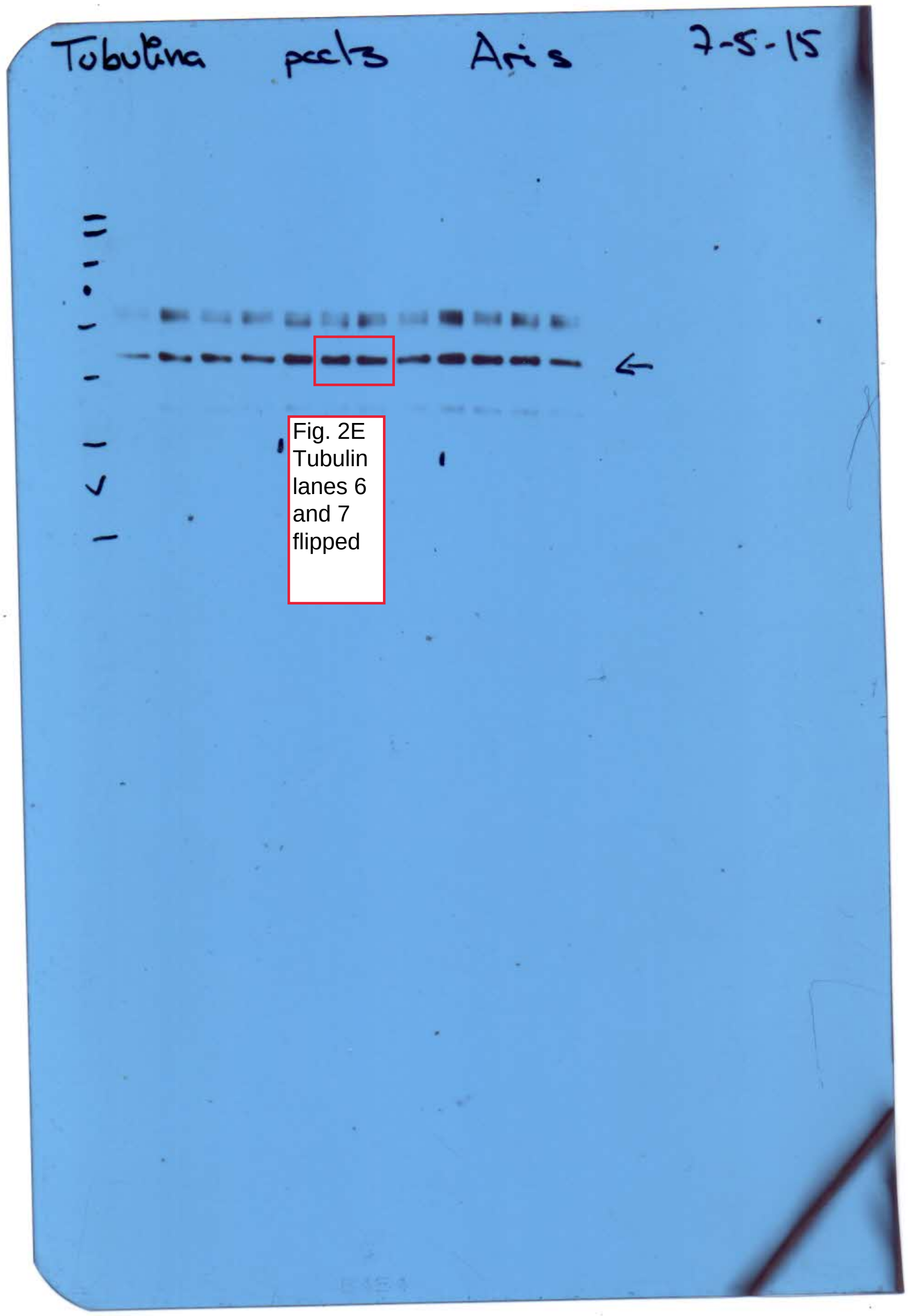
Tubulina
pccl3
Aris
7-5-15
Fig. 2E Tubulin lanes 6 and 7 flipped

Fig. 3B Sox9
Lanes1-4

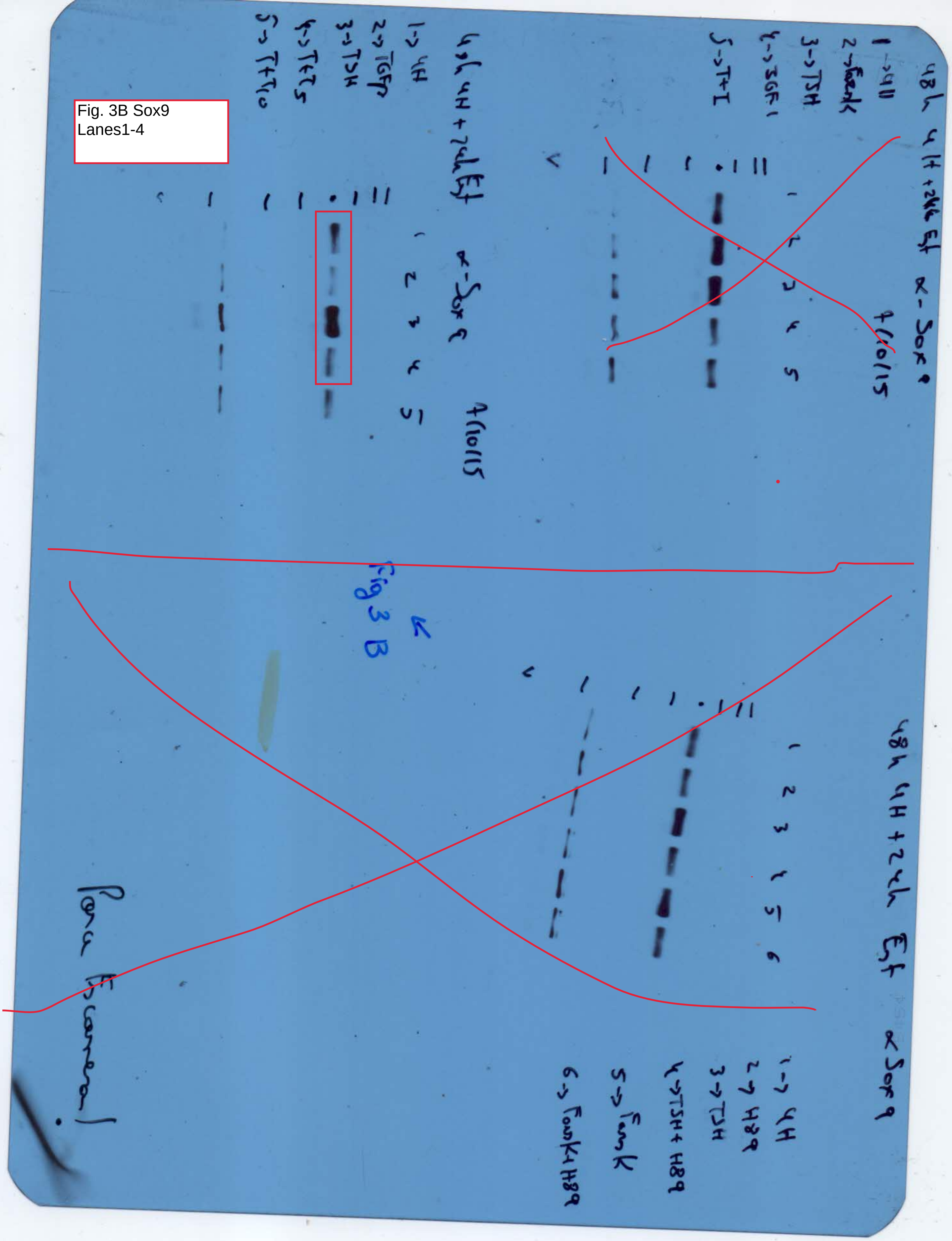

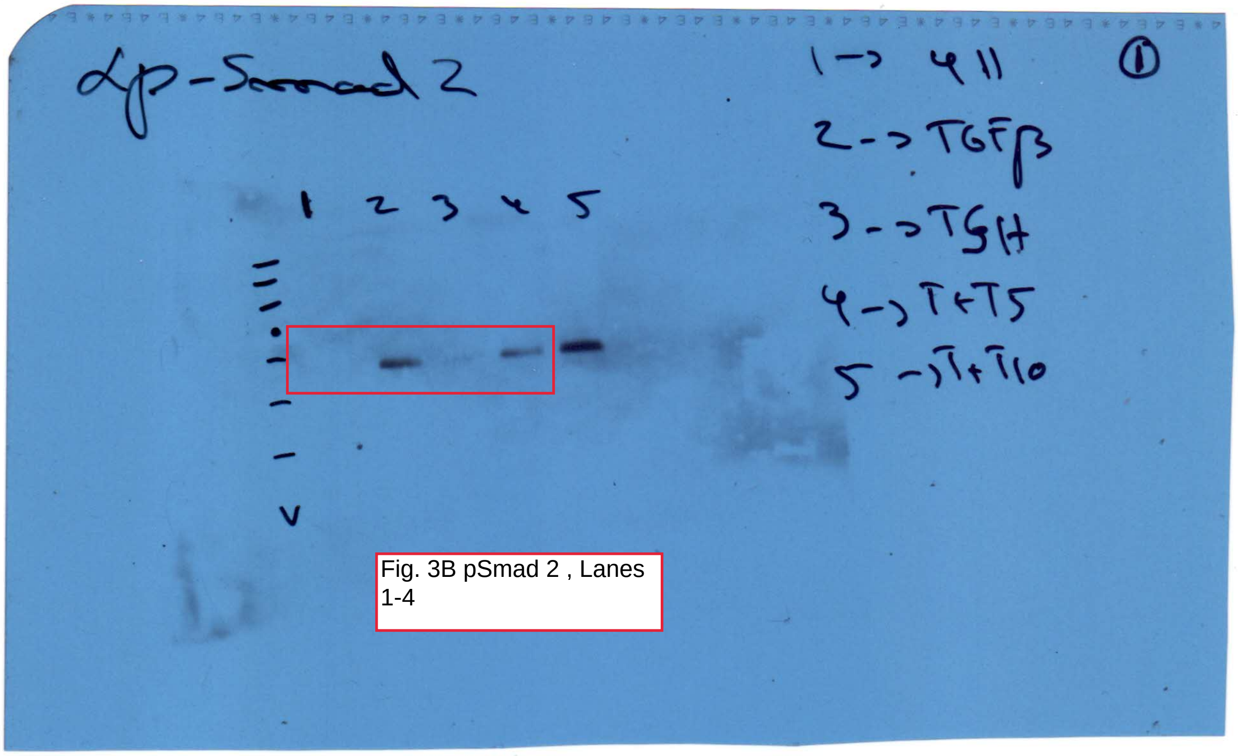

Fig. 3B pSmad 2 , Lanes 1-4

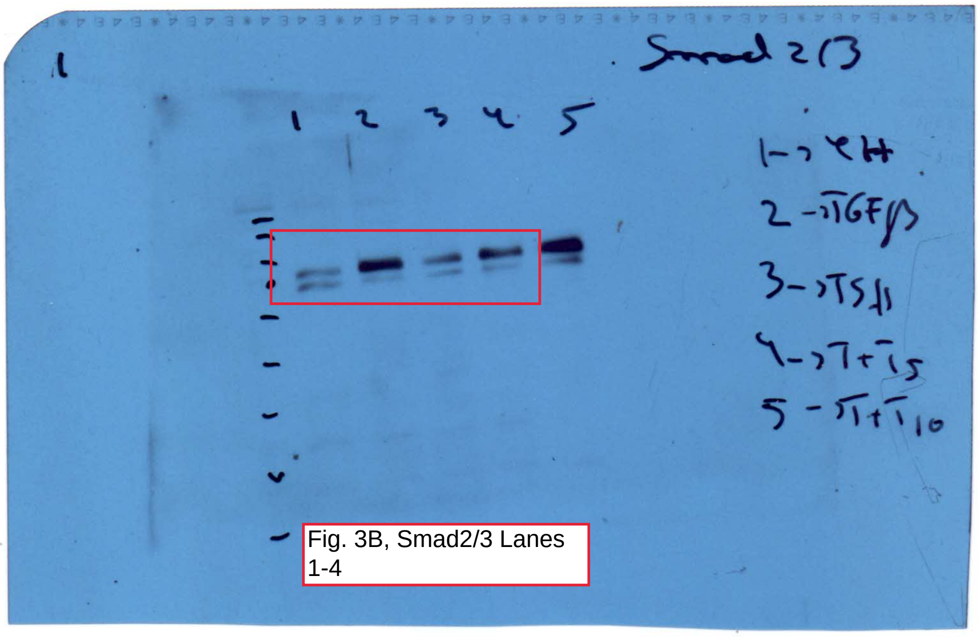

Fig. 3B, Smad2/3 Lanes 1-4

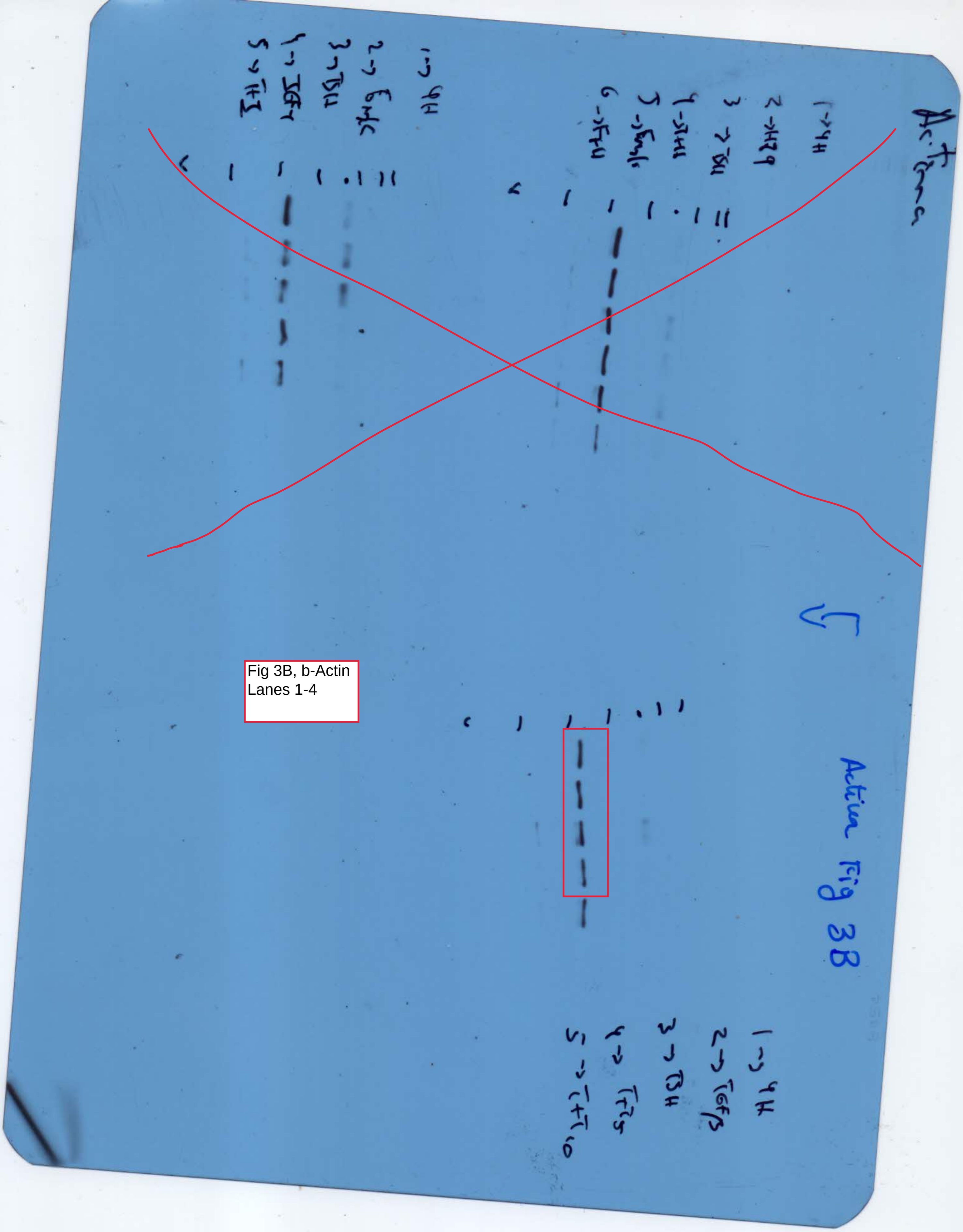

Fig 3B, b-Actin
Lanes 1-4

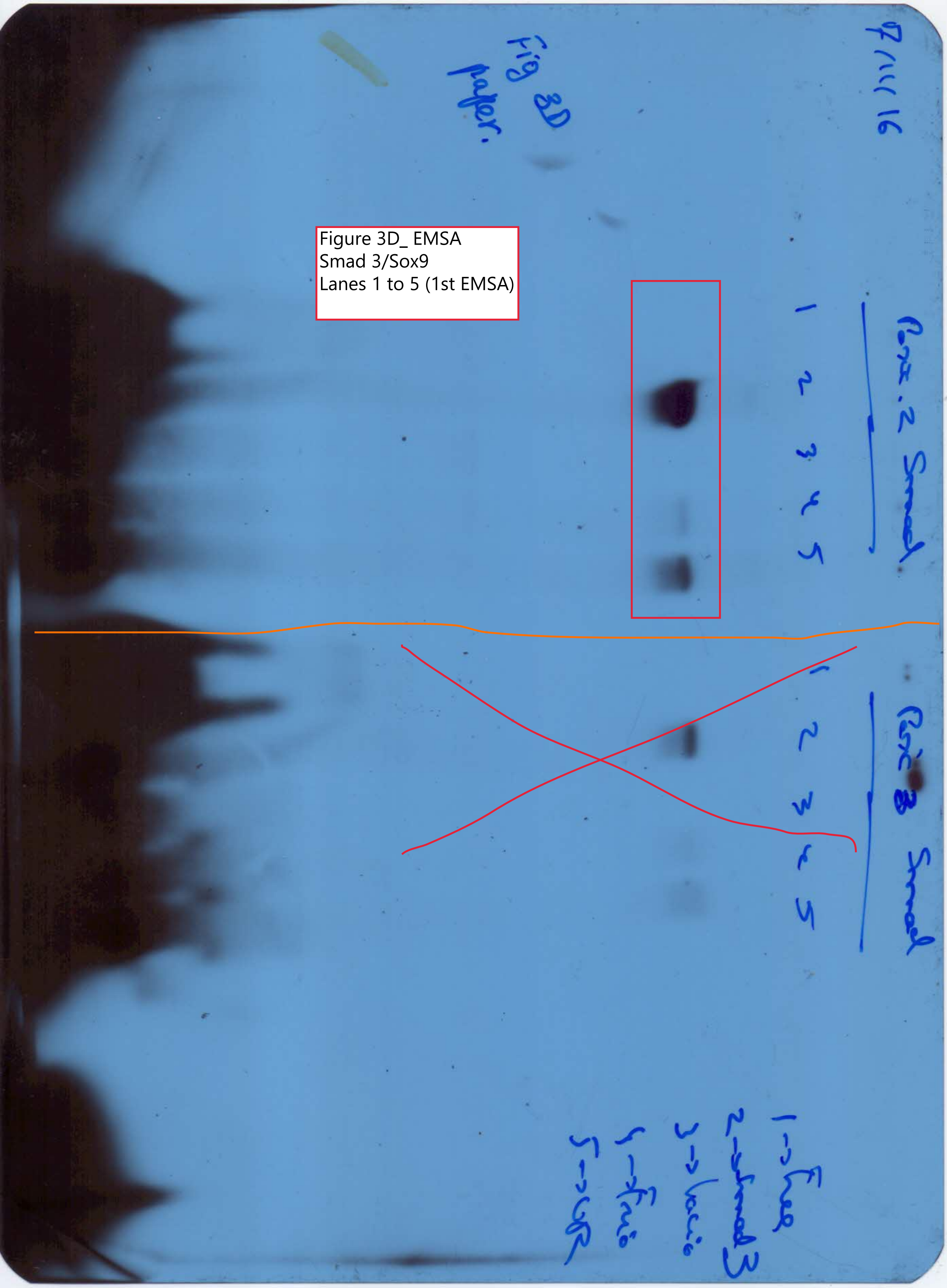
Figure 3D_ EMSA
Smad 3/Sox9
Lanes 1 to 5 (1st EMSA)

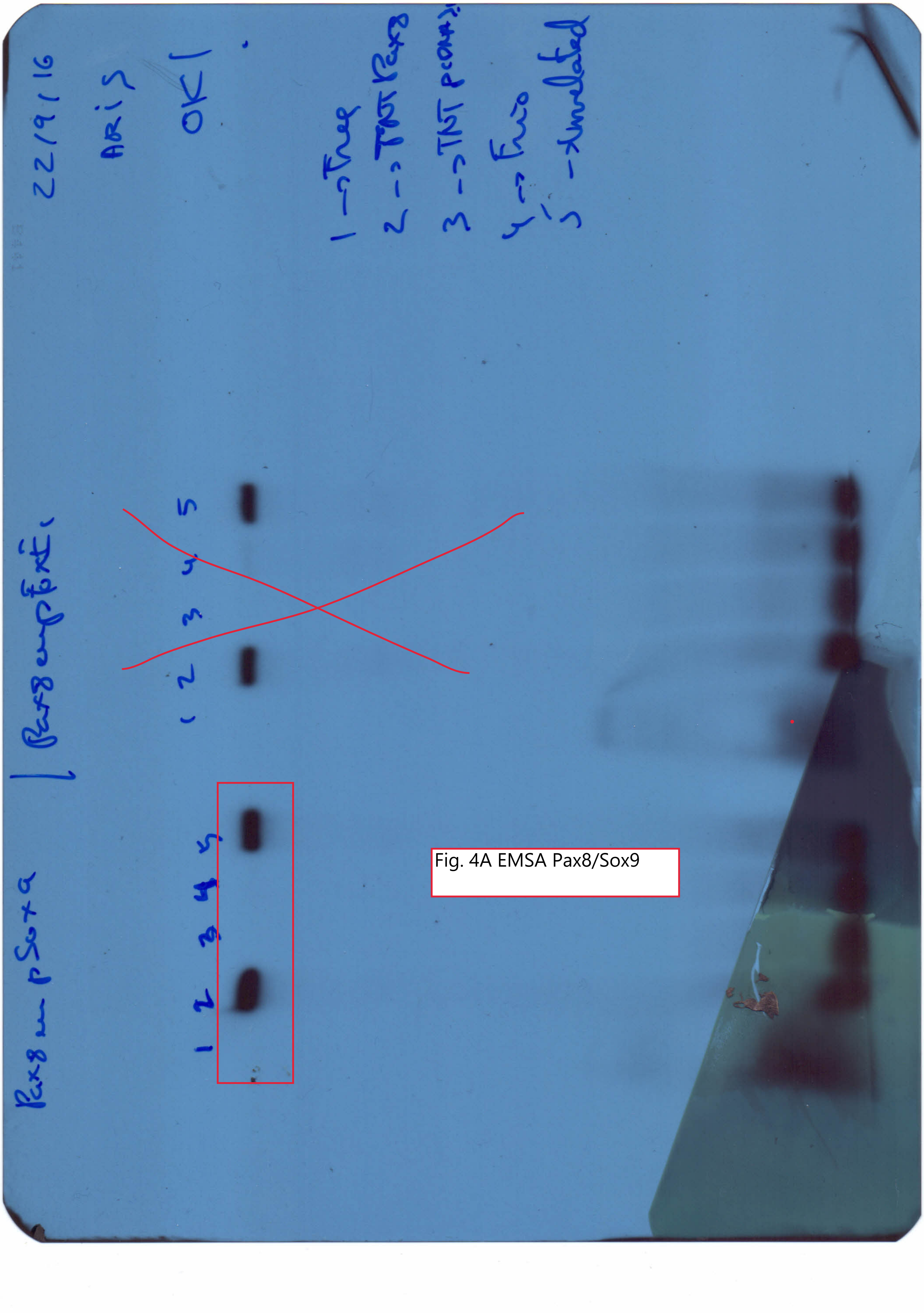

Fig. 4A EMSA Pax8/Sox9

Fig. 4B EMSA Foxe1/Sox9

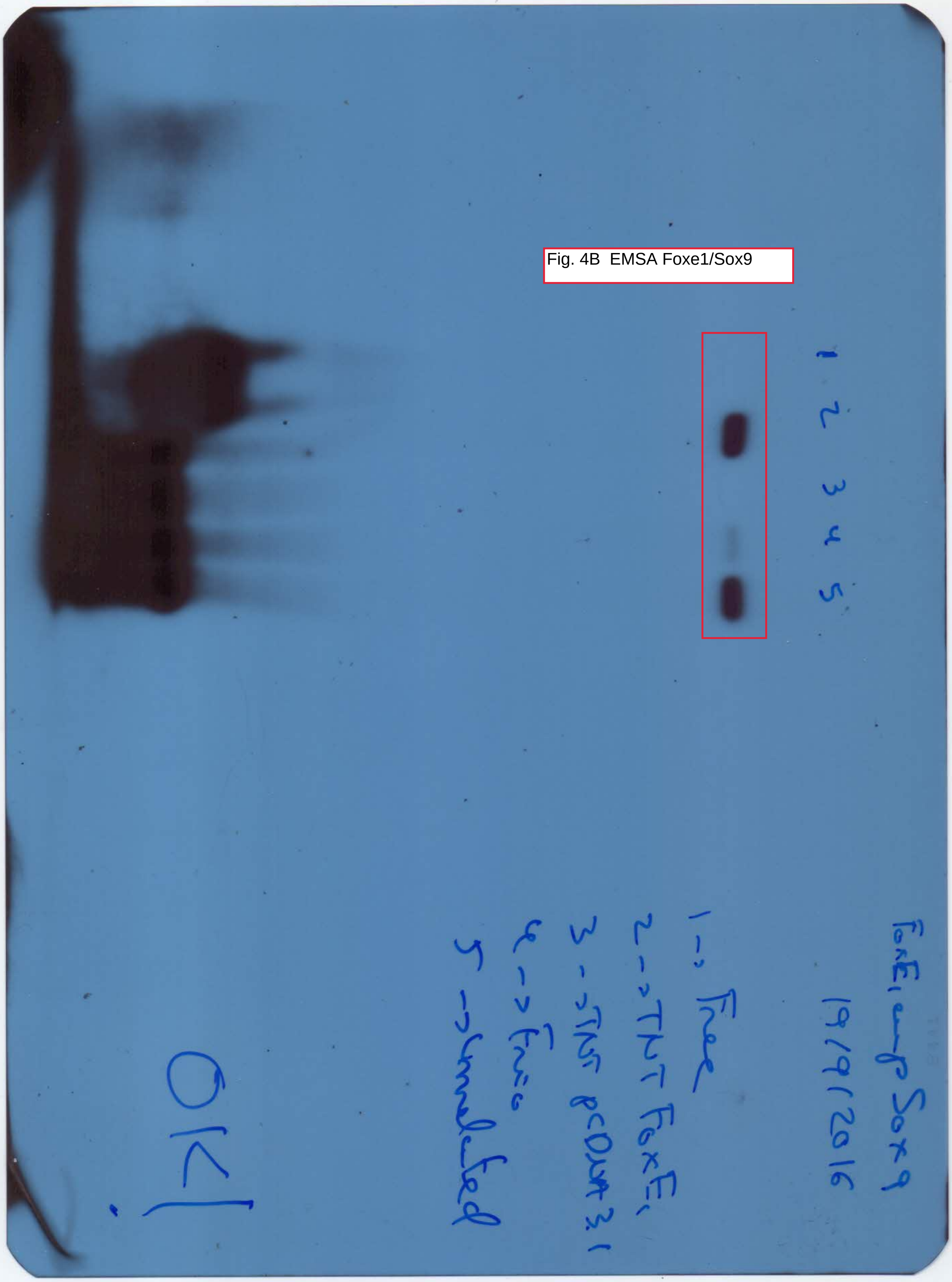

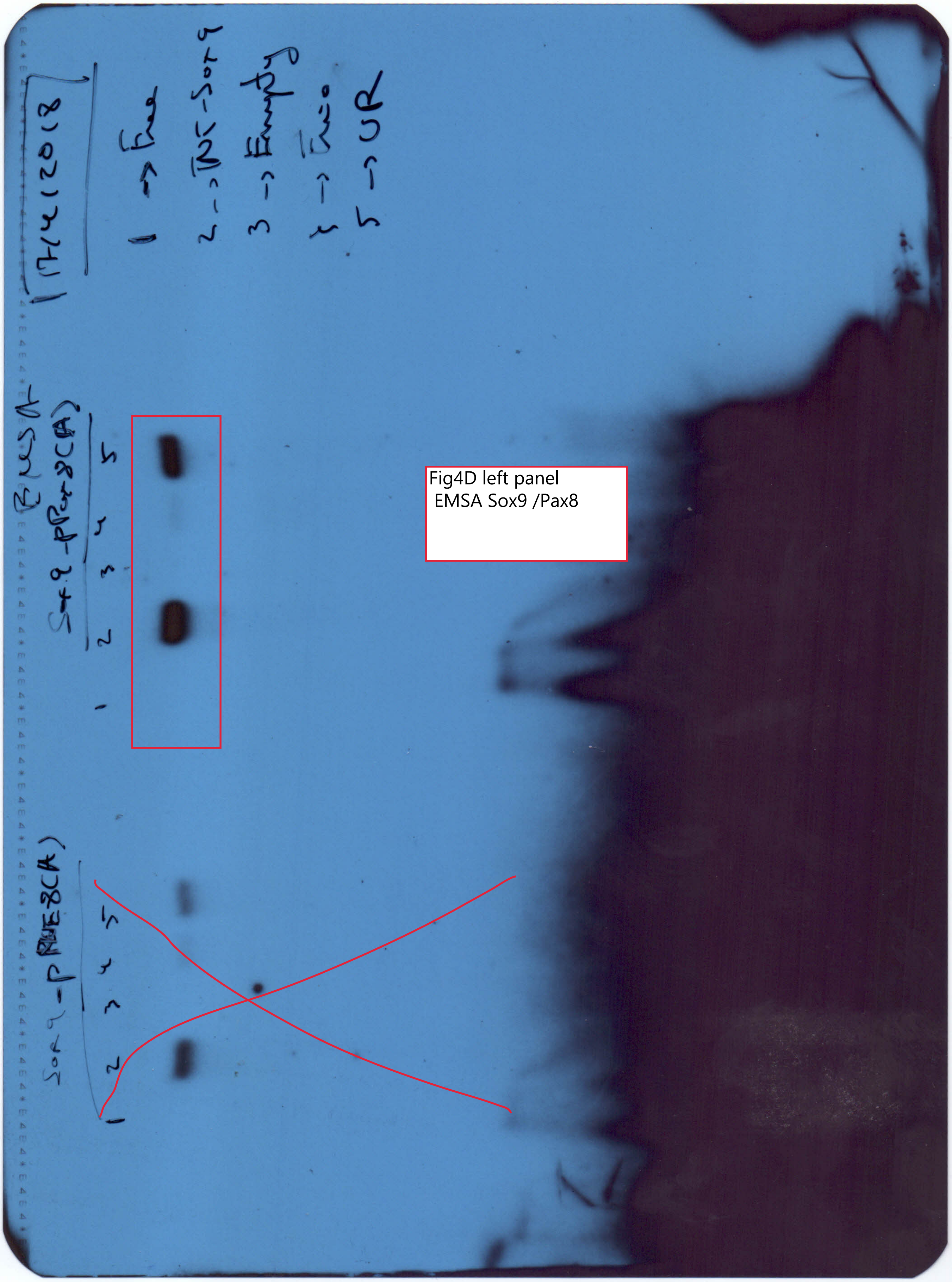

Fig4D left panel
EMSA Sox9 /Pax8

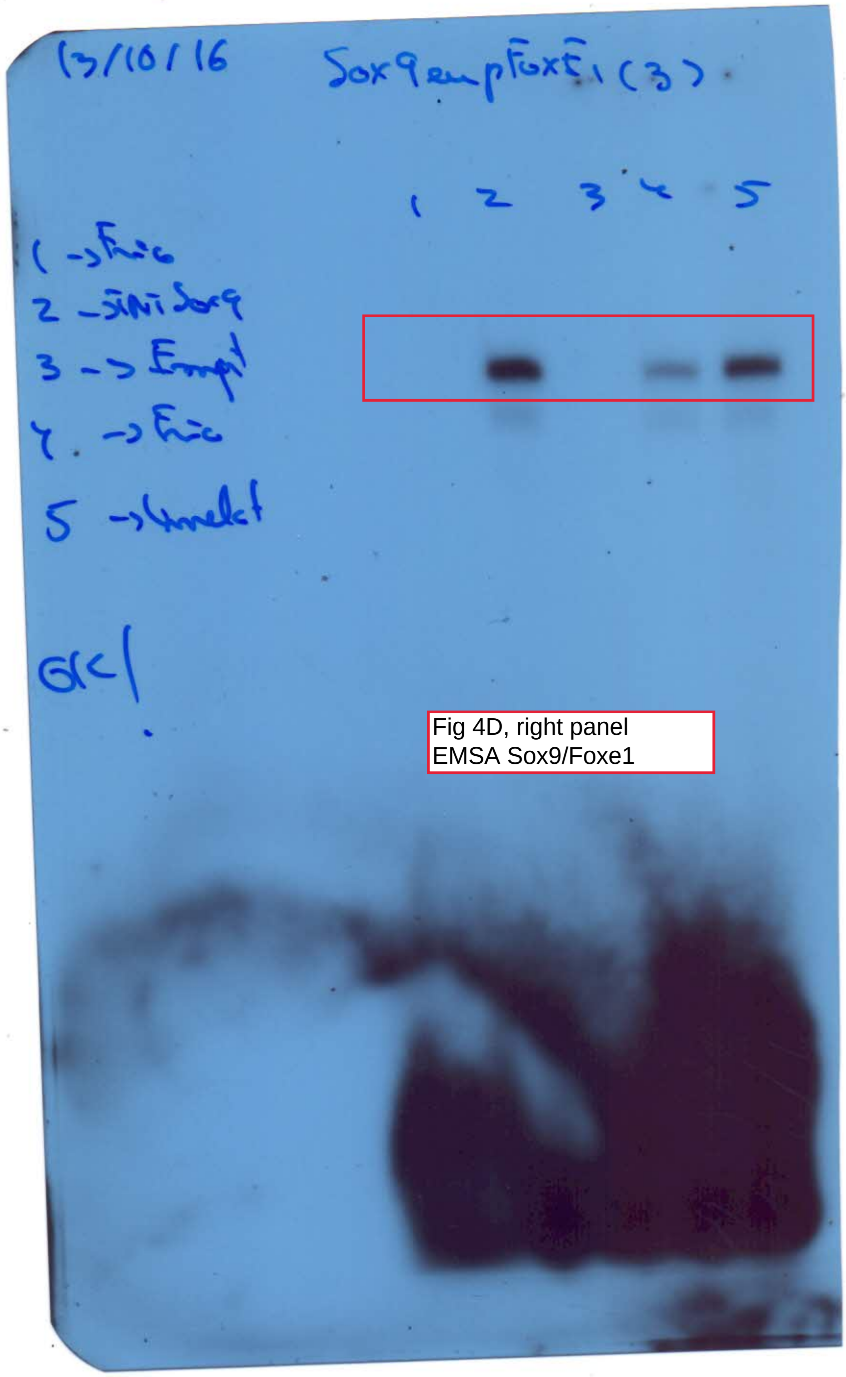

Fig 4D, right panel
EMSA Sox9/Foxe1

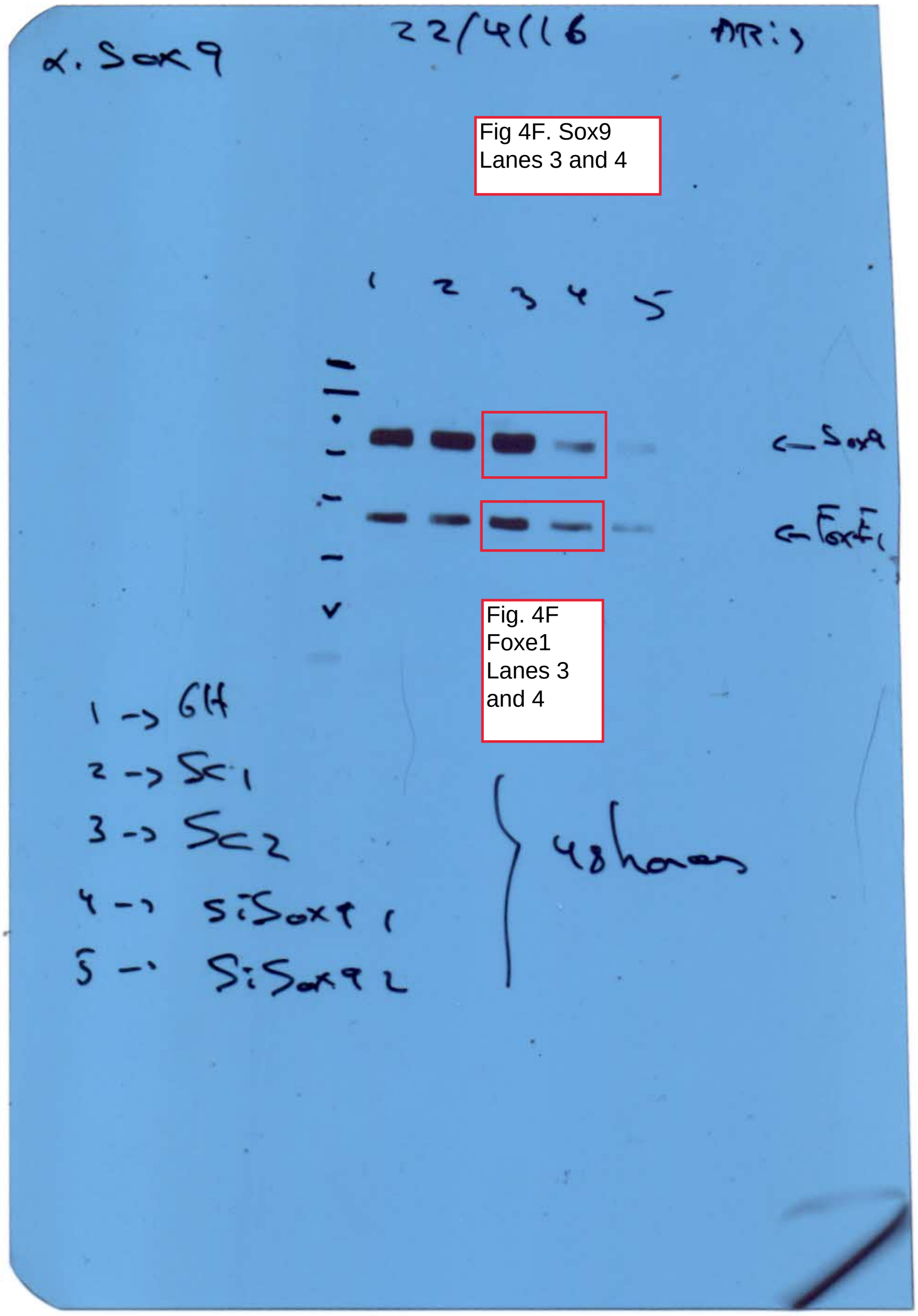

α. Sox9
22/4/16
Fig 4F. Sox9
Lanes 3 and 4
← Sox9
← FoxE1
Fig. 4F
Foxe1
Lanes 3
and 4
2 -> Sc1
3 -> Sc2
4 -> siSox9 1
5 -> siSox9 2
48hours

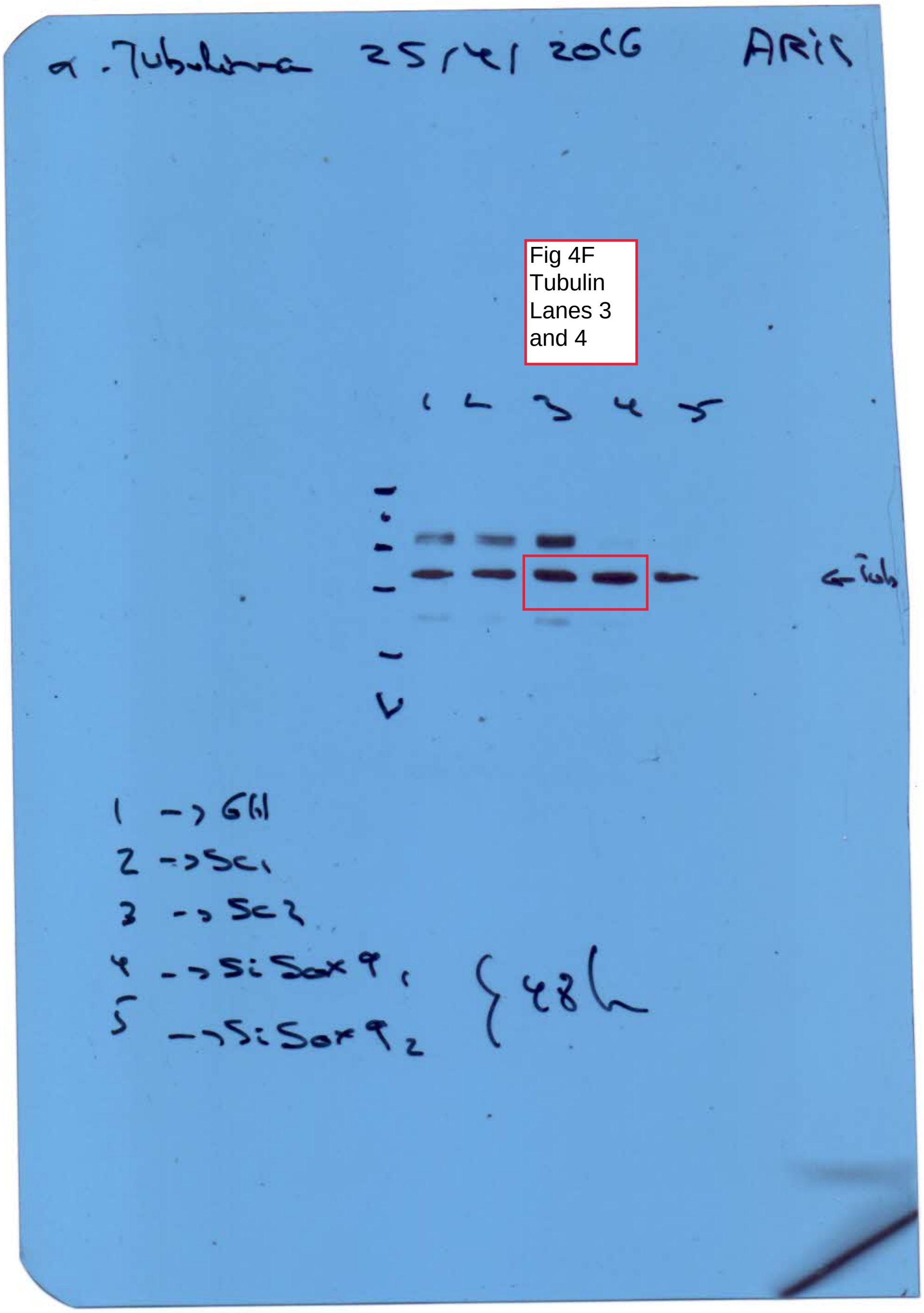
Fig 4F
Tubulin
Lanes 3
and 4
1 2 3 4 5
2 -> Sc1
3 -> Sc2
4 -> siSox9 1
5 -> siSox9 2
48h

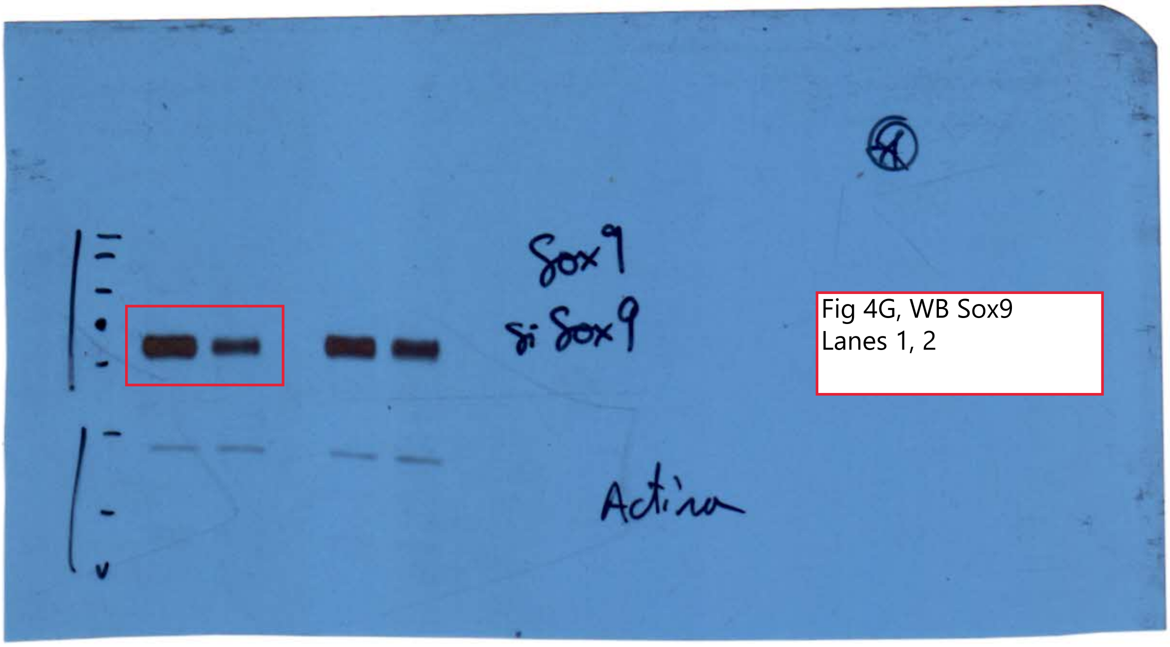
Sox9
si Sox9
Fig 4G, WB Sox9
Lanes 1, 2
Actin

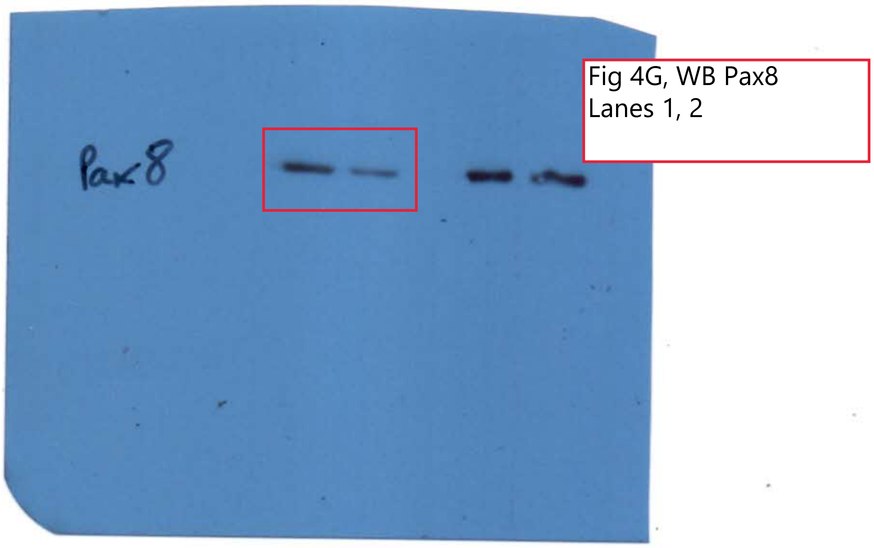
Fig 4G, WB Pax8
Lanes 1, 2
Pax8

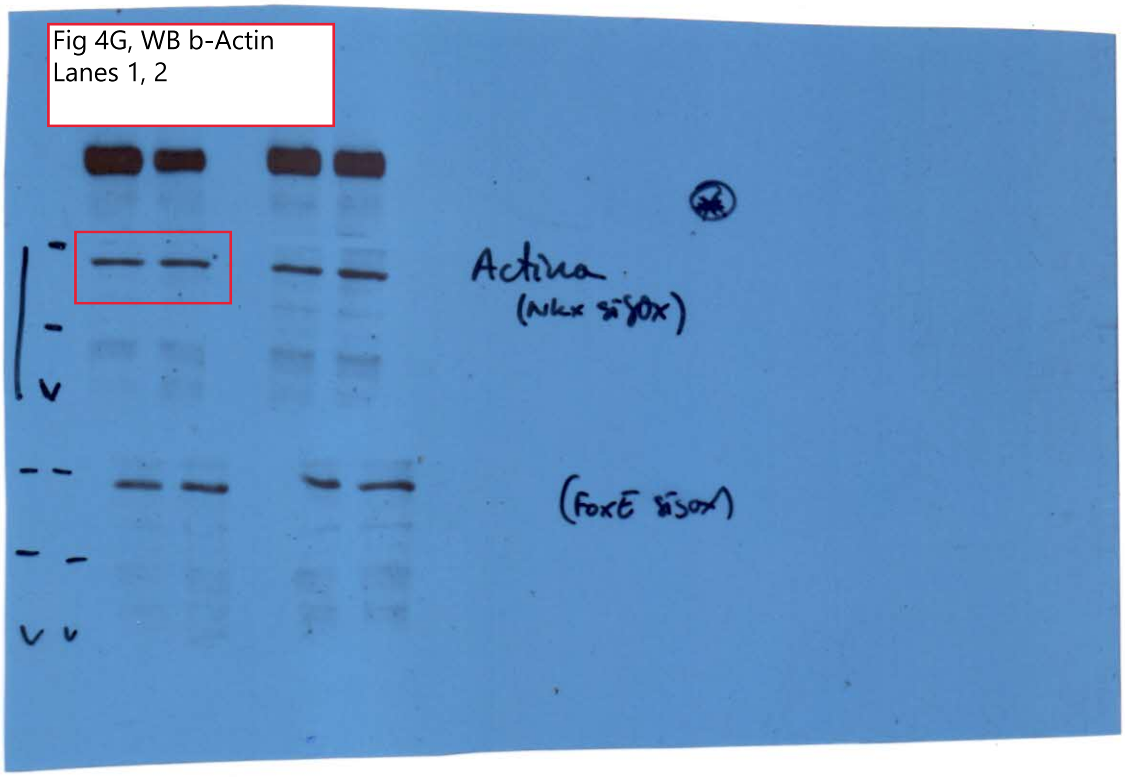
Fig 4G, WB b-Actin
Lanes 1, 2
Actin

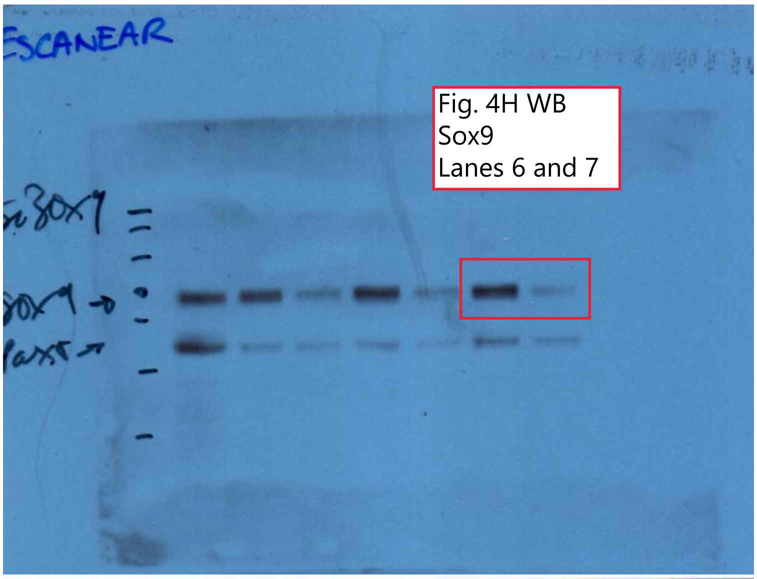
ESCANEAR
Fig. 4H WB
Sox9
Lanes 6 and 7

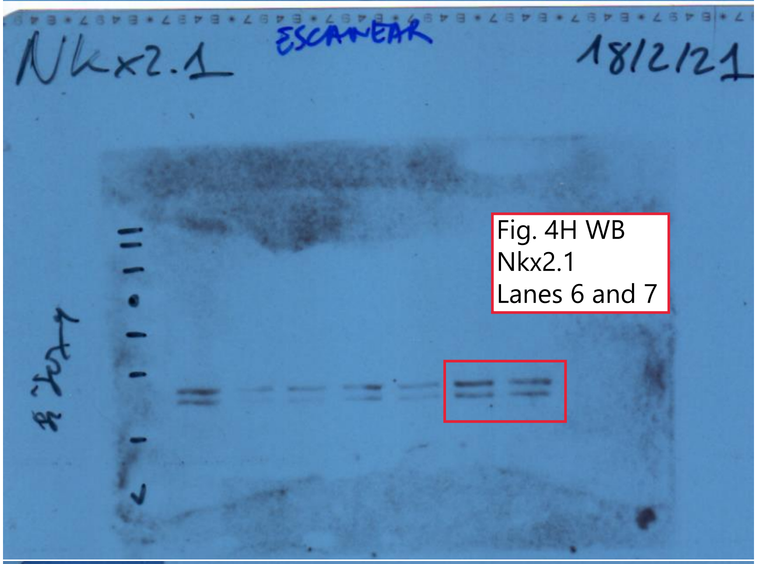
Nkx2.1
ESCANEAR
18/12/21
Fig. 4H WB
Nkx2.1
Lanes 6 and 7

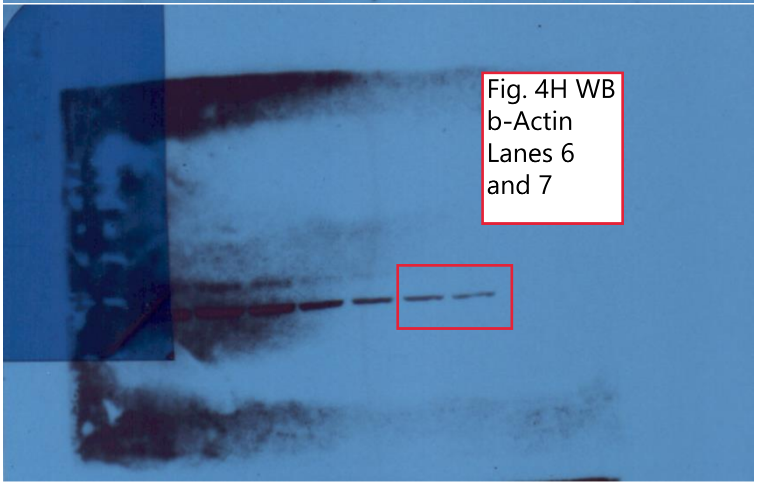
Fig. 4H WB
b-Actin
Lanes 6
and 7

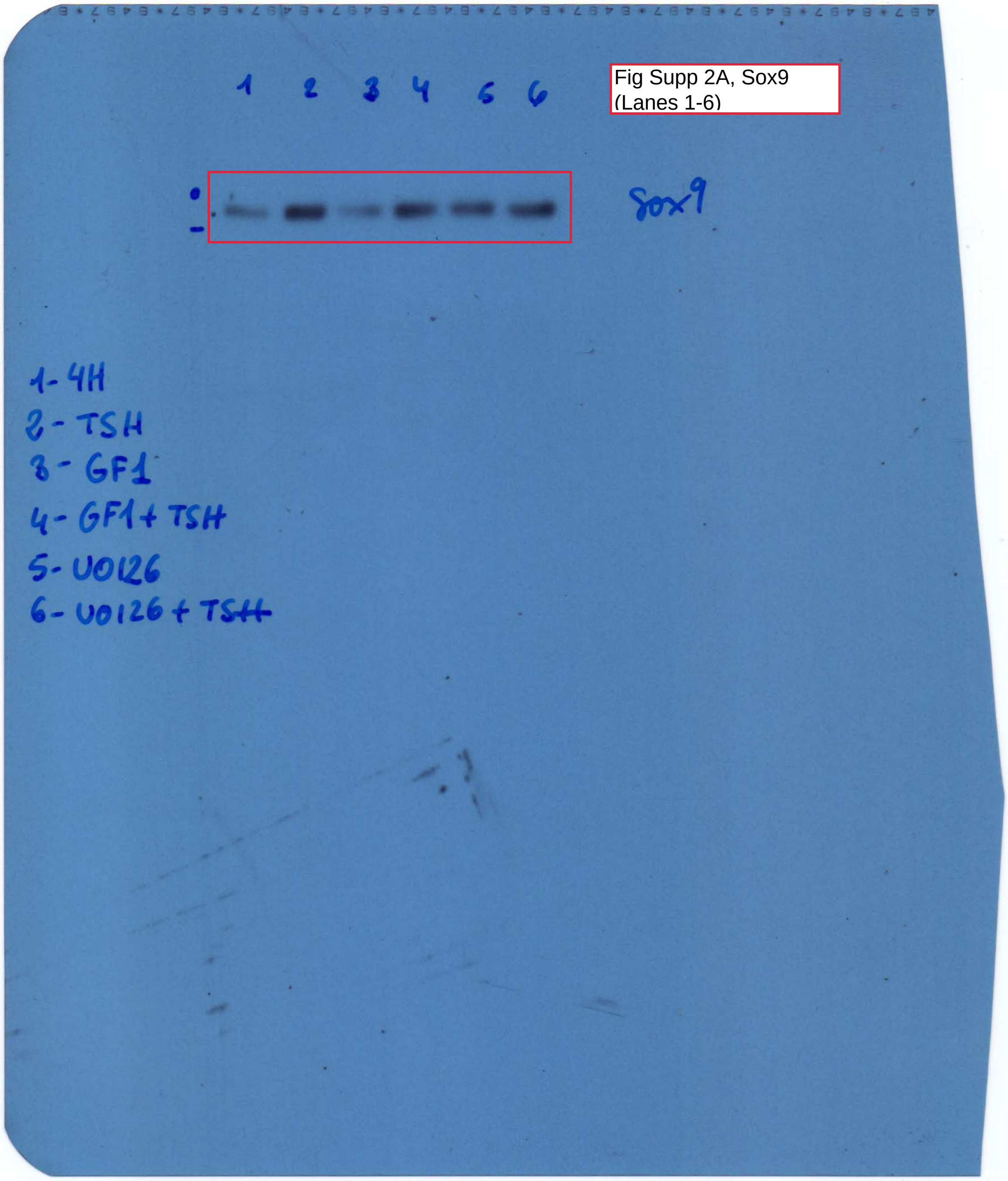
1 2 3 4 5 6
Fig Supp 2A, Sox9
(Lanes 1-6)
Sox9
1- 4H
2- TSH
3- GF1
4- GF1 + TSH
5- U0126
6- U0126 + TSH

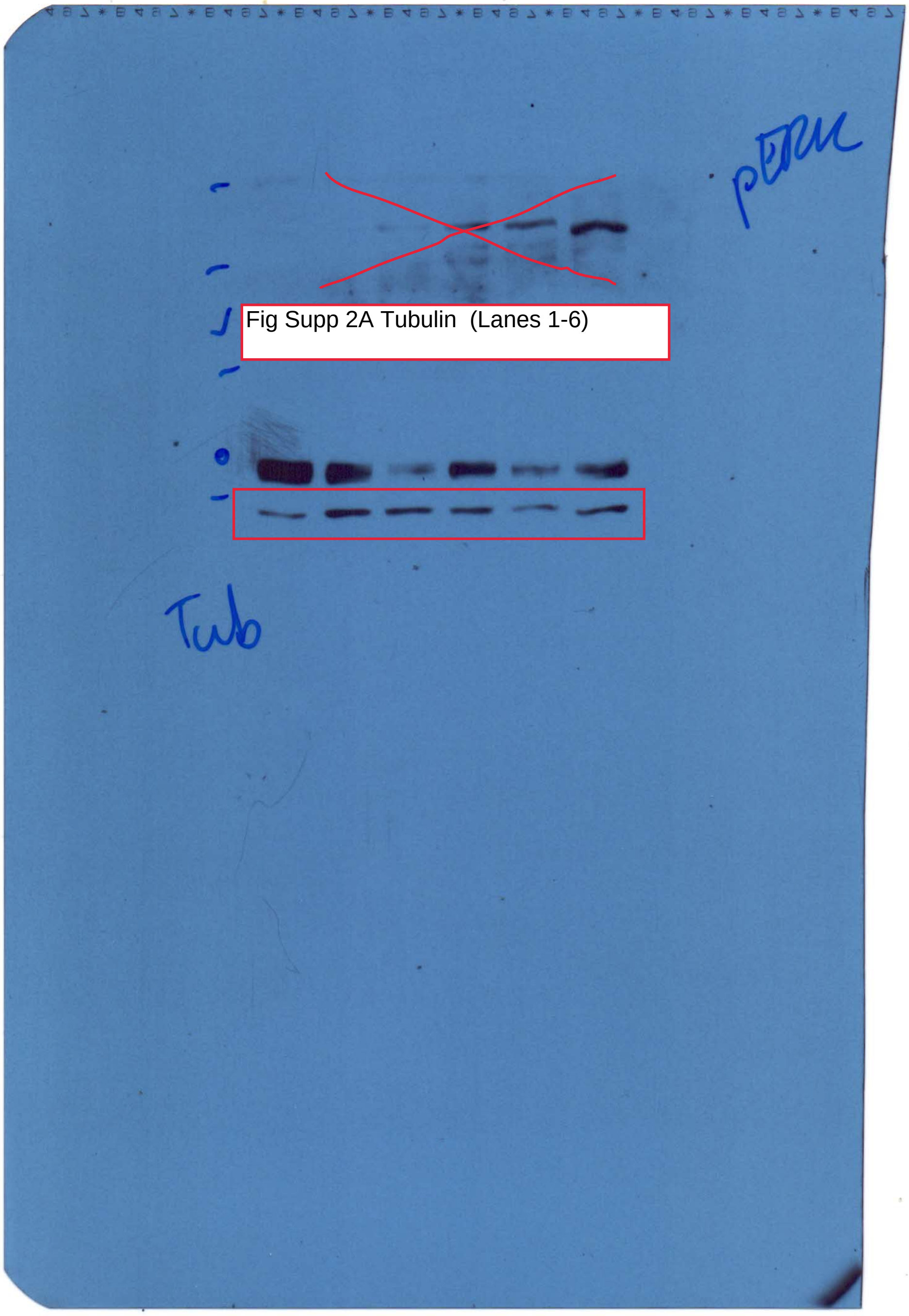

Fig Supp 2A Tubulin (Lanes 1-6)

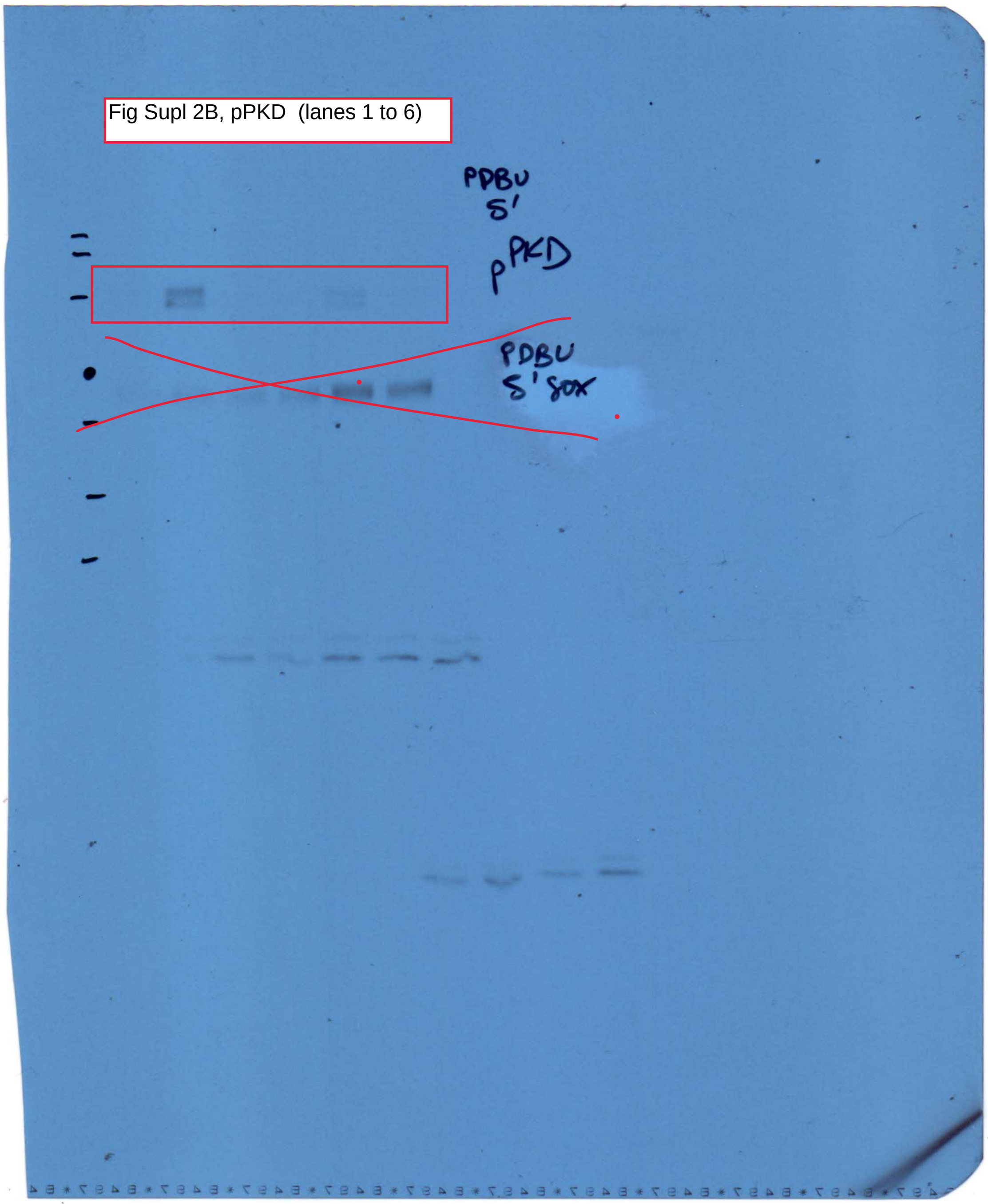
Fig Supl 2B, pPKD (lanes 1 to 6)
PDBU 5'
pPKD
PDBU 5' sox

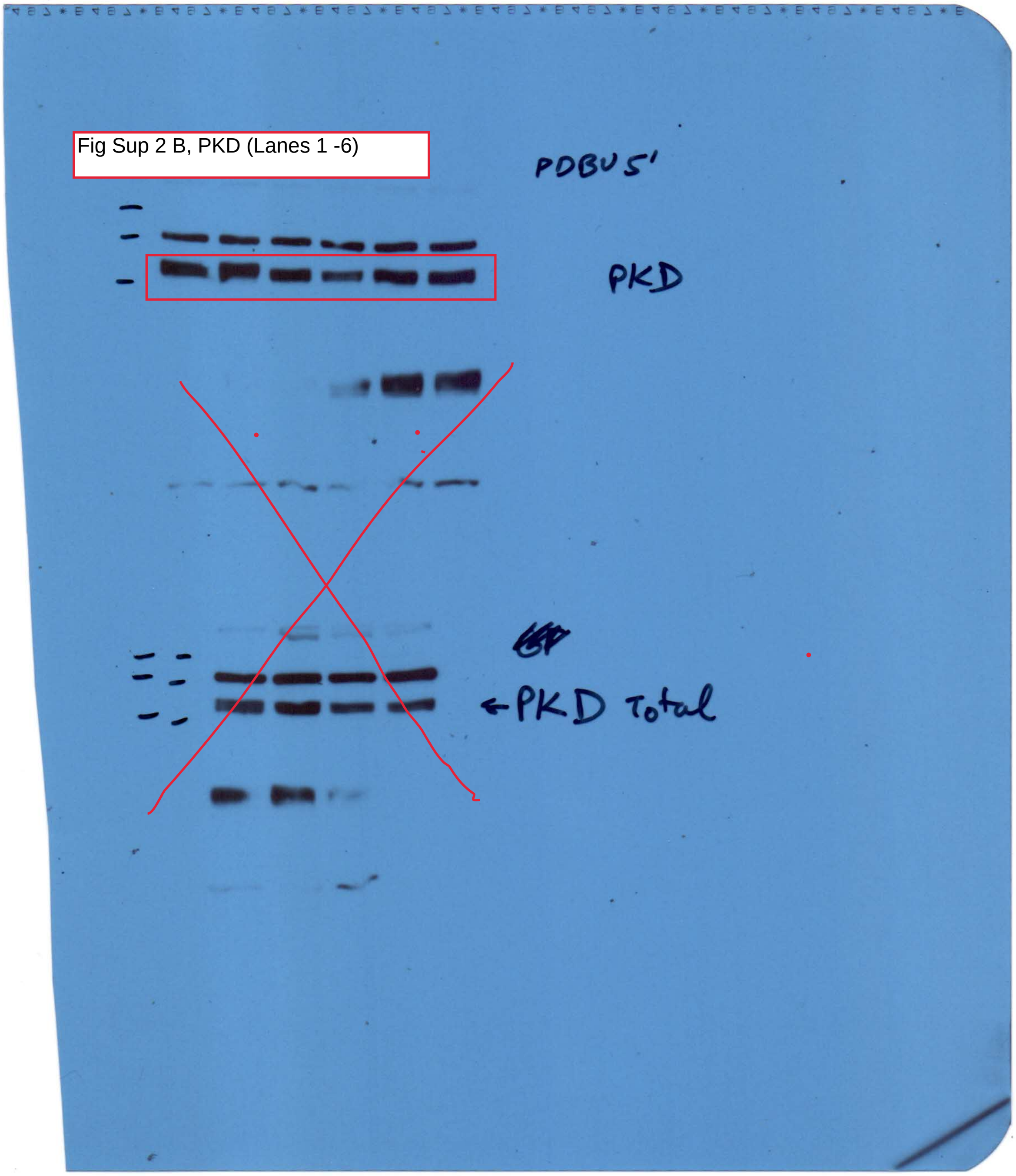
Fig Sup 2 B, PKD (Lanes 1 -6)
PDBU 5'
PKD
←PKD Total

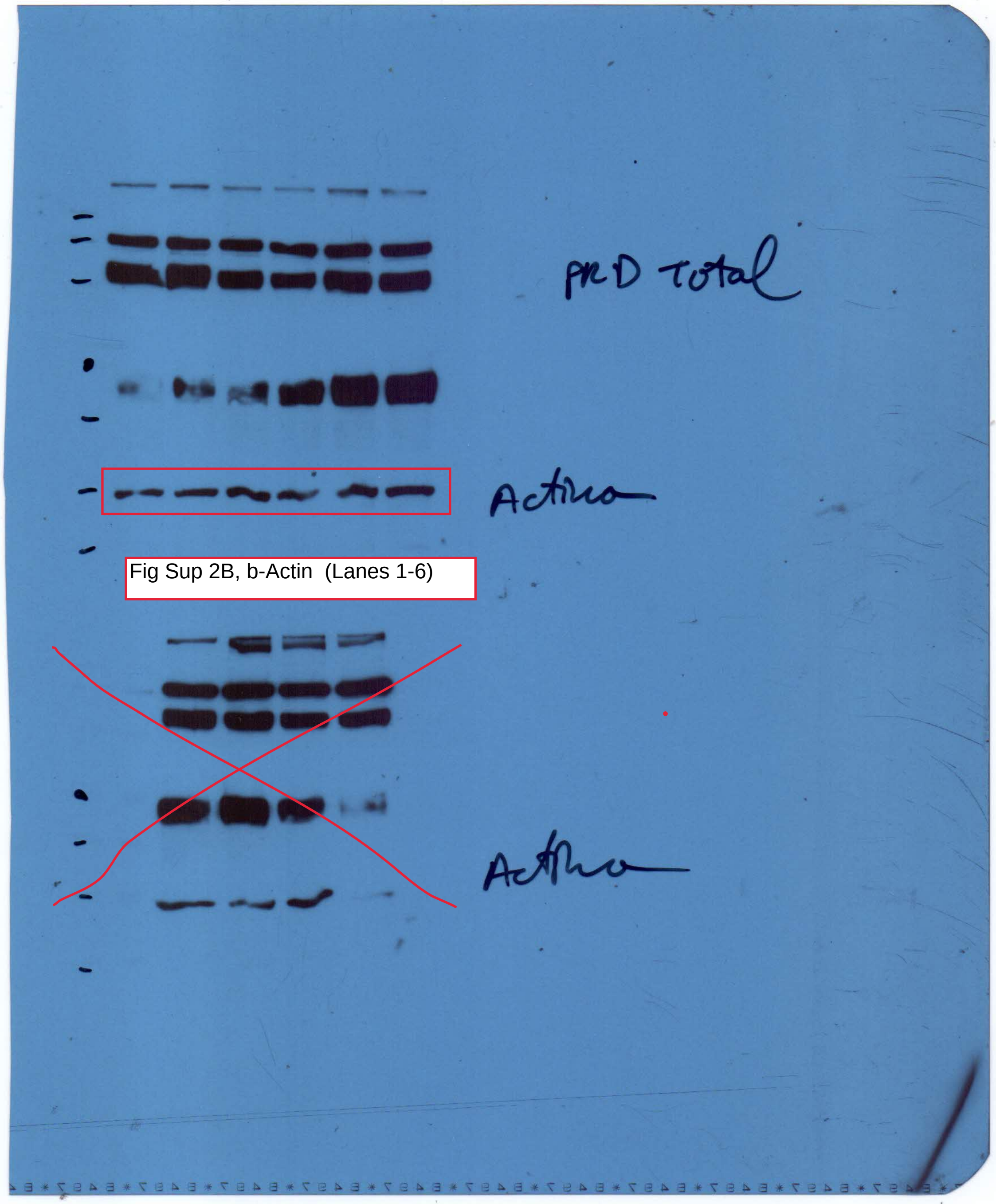
Actin
Fig Sup 2B, b-Actin (Lanes 1-6)
Actin

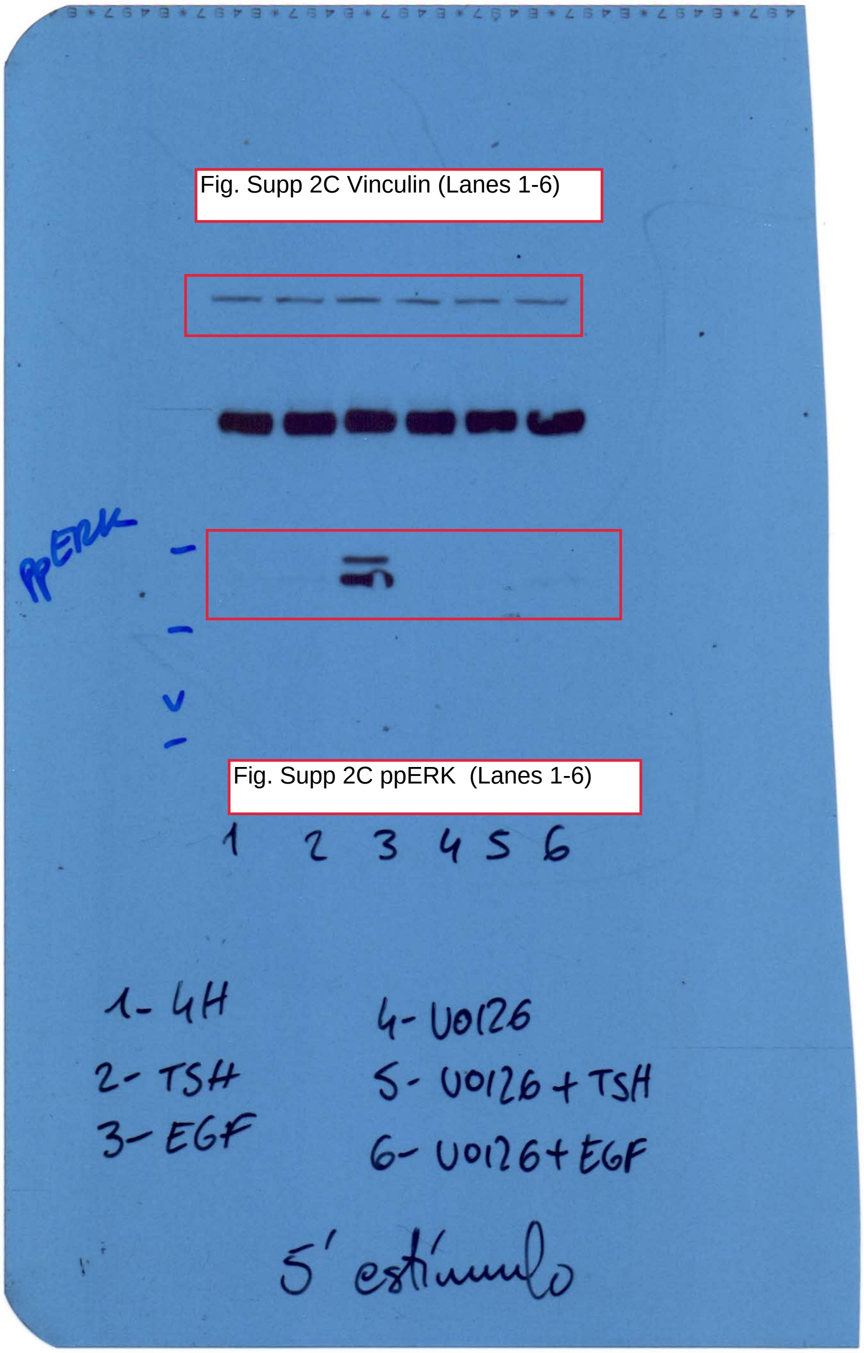
Fig. Supp 2C Vinculin (Lanes 1-6)
ppERK
Fig. Supp 2C ppERK (Lanes 1-6)
1 2 3 4 5 6
1- 4H
2- TSH
3- EGF
4- U0126
5- U0126 + TSH
6- U0126+EGF
5' estímulo

Fig. Supp 2C ERK (Lanes 1-6)

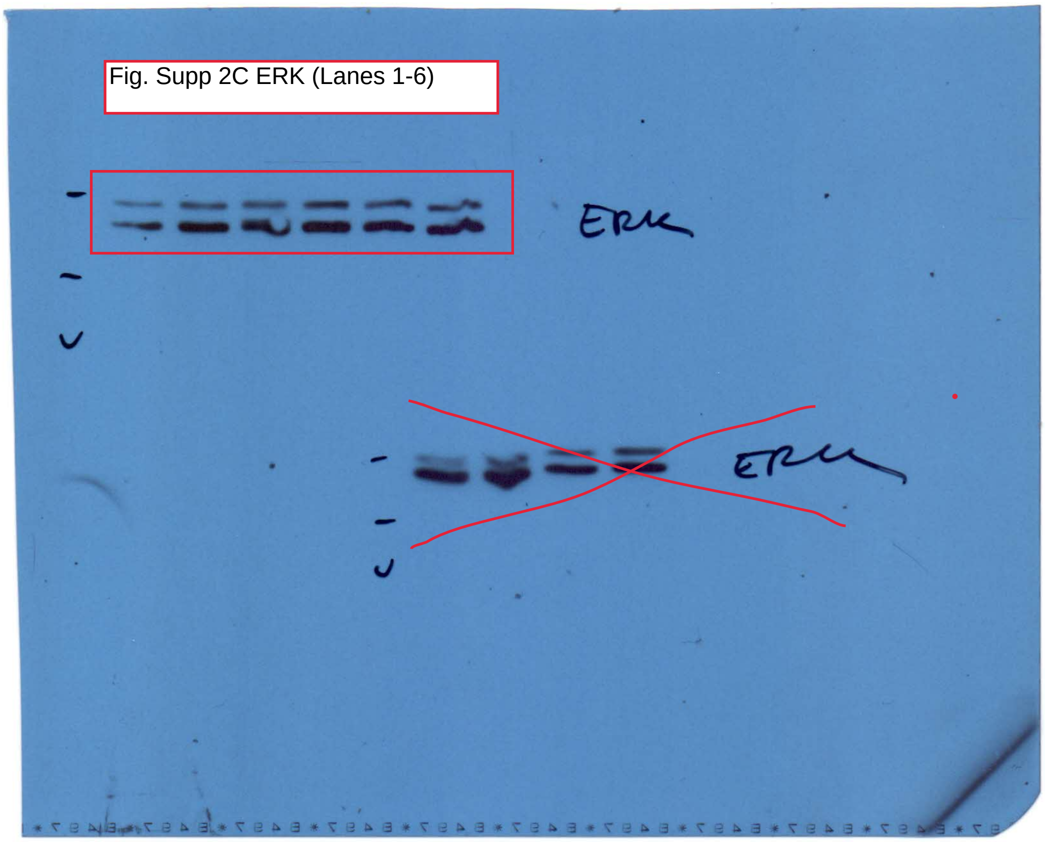

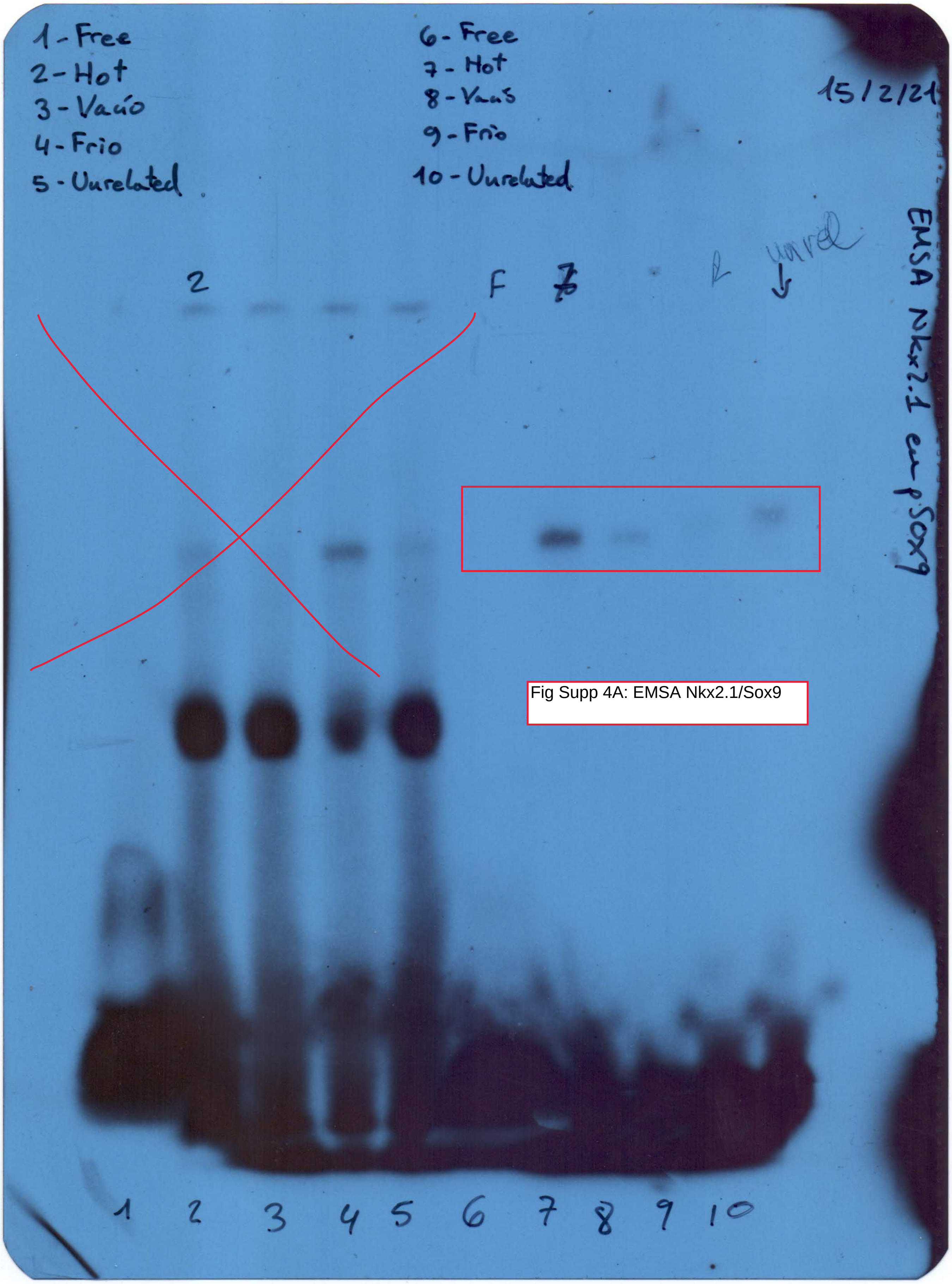

Fig Supp 4A: EMSA Nkx2.1/Sox9

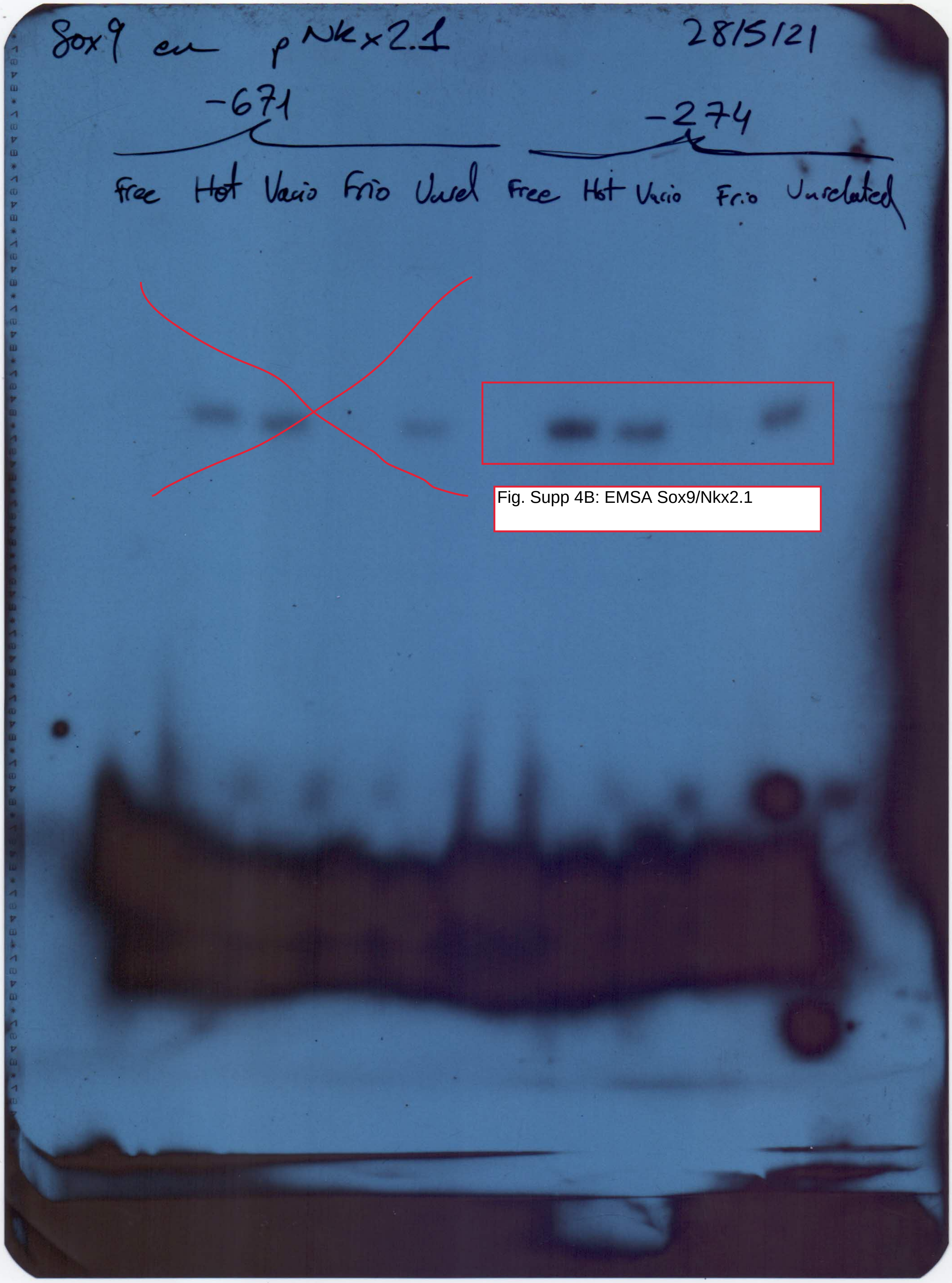

Fig. Supp 4B: EMSA Sox9/Nkx2.1
