# Supplementary material for: Sox9 is involved in the thyroid differentiation program and is regulated by crosstalk between TSH, TGFβ and thyroid transcription factors
Source: Sci Rep. 2022 Feb 9;12:2144. doi: 10.1038/s41598-022-06004-1 (PMC8828901; doi:10.1038/s41598-022-06004-1)
Supplement: Supplementary file 6 — Supplementary Information 6. [file 41598_2022_6004_MOESM6_ESM.docx]

| **Gene** | **Oligonucleotide sequence** |
| --- | --- |
| *β-Actin* | Forward: 5´- CTCTCTTCCAGCCTTCCTT–3’ |
|  | Reverse: 5´- CTCATCGTACTCCTGCTTGCT–3’ |
| *Sox9* | Forward: 5´- TACGACTGGACCCTGGTG –3’ |
|  | Reverse: 5´- AGATGTGAGTCTGTTCGGTG –3’ |

Supplementary Table 1. Oligonucleotides used for RT-qPCR.

| **Protein** | **Company** | **Reference** | **Use** |
| --- | --- | --- | --- |
| Sox9 | Merck-Millipore | AB-5535 | WB, IF, IH |
| CREB | Santa Cruz Biotechnology | sc-186 | WB |
| Foxe1 | Biopat | PA0200 | WB |
| Pax8 | Biopat | PA0300 | WB |
| Nkx2.1 | Biopat | PA0100 | IF, WB |
| pSmad2 | Abcam | ab53100 | WB |
| Smad2/3 | Abcam | ab217553 | WB |
| βActin | Santa Cruz Biotechnology | sc-1616R | WB |
| Nkx2.1 | Dako | M3575 | IF |
| GFP | Abcam | 6673 | IF |
| PKD | Invitrogen | PA5-78126 | WB |
| pPKD | Cell Signaling | #2051 | WB |
| ERK | Santa Cruz Biotechnology | sc-514302 | WB |
| ppERK | Cell Signaling | #4370 | WB |
| Vinculin | Santa Cruz | sc-73614 | WB |
| Tubulin | Santa Cruz Biotechnology | sc-5286 | WB |
| E-Cadherin | BD Biosciences | 610182 | IF |

WB, western blot; IF, immunofluorescence; IH, immunohistochemistry

Supplementary Table 2. Antibodies used for protein detection.

| **Transcription factor** | **Sox9 promoter sequence** |
| --- | --- |
| Foxe1 | 5’-TGAGTCACCCAAACATTTCTTCCA-3’ |
|  | 5’-TGGAAGAAATGTTTGGGTGACTCA-3’ |
| Pax8 | 5’-TTTCGGTCCAGGAACTTTTCTTTG-3’ |
|  | 5’-CAAAGAAAAGTTCCTGGACCGAAA -3’ |
| Nkx2.1 | 5’- GTCTGGAAACTTCAGTGGGAGCGA-3’ |
|  | 5’- TCGCTCCCACTGAAGTTTCCAGAC-3’ |
| CREB | 5´-CCTCGAGTCTCGTCACCCA-3´ |
|  | 5´-TGGGTGACGAGACTCGAGG-3’ |
| Smad3 | 5’-CTCGGAACTGTCTGGAAACTTCAG-3’ |
|  | 5’-CTGAAGTTTCCAGACAGTTCCGAG-3’ |
| **Gene** | **Sox9 binding sequence** |
| Foxe1 | 5´-TCATGACCTTTGTATTAATC-3´ |
|  | 5´-GATTAATACAAAGGTCATGA-3´ |
| Pax8 | 5’- GGCCCTCAGAACAAAGTTCTCACT-3’ |
|  | 5’- AGTGAGAACTTTGTTCTGAGGGCC-3’ |
| Nkx2.1 | 5’- AGGCTTCTATTGTCTTGTTGCTTT-3’ |
|  | 5’- AAAGCAACAAGACAATAGAAGCCT-3’ |

**Supplementary Table 3. Oligonucleotides containing the transcription factor binding sites used for electrophoresis mobility shift assays.**
